# Magnetotactic Bacteria Accumulate a Large Pool of Iron Distinct from Their Magnetite Crystals

**DOI:** 10.1128/AEM.01278-20

**Published:** 2020-10-28

**Authors:** Matthieu Amor, Alejandro Ceballos, Juan Wan, Christian P. Simon, Allegra T. Aron, Christopher J. Chang, Frances Hellman, Arash Komeili

**Affiliations:** a Department of Plant and Microbial Biology, University of California, Berkeley, California, USA; b Department of Materials Science and Engineering, University of California, Berkeley, California, USA; c Department of Physics, University of California, Berkeley, California, USA; d Department of Chemistry, University of California, Berkeley, California, USA; e Skaggs School of Pharmacy and Pharmaceutical Sciences, University of California, San Diego, La Jolla, California, USA; f Collaborative Mass Spectrometry Innovation Center, University of California, San Diego, La Jolla, California, USA; g Department of Molecular and Cell Biology, University of California, Berkeley, California, USA; h Materials Sciences Division, Lawrence Berkeley National Laboratory, Berkeley, California, USA; University of Tennessee at Knoxville

**Keywords:** biomineralization, iron, magnetite, magnetotactic bacteria

## Abstract

Magnetotactic bacteria (MTB) are ubiquitous aquatic microorganisms that form intracellular nanoparticles of magnetite (Fe_3_O_4_) or greigite (Fe_3_S_4_) in a genetically controlled manner. Magnetite and greigite synthesis requires MTB to transport a large amount of iron from the environment. Most intracellular iron was proposed to be contained within the crystals. However, recent mass spectrometry studies suggest that MTB may contain a large amount of iron that is not precipitated in crystals. Here, we attempted to resolve these discrepancies by performing chemical and magnetic assays to quantify the different iron pools in the magnetite-forming strain Magnetospirillum magneticum AMB-1, as well as in mutant strains showing defects in crystal precipitation, cultivated at various iron concentrations. All results show that magnetite represents at most 30% of the total intracellular iron under our experimental conditions and even less in the mutant strains. We further examined the iron speciation and subcellular localization in AMB-1 using the fluorescent indicator FIP-1, which was designed for the detection of labile Fe(II). Staining with this probe suggests that unmineralized reduced iron is found in the cytoplasm and associated with magnetosomes. Our results demonstrate that, under our experimental conditions, AMB-1 is able to accumulate a large pool of iron distinct from magnetite. Finally, we discuss the biochemical and geochemical implications of these results.

**IMPORTANCE** Magnetotactic bacteria (MTB) produce iron-based intracellular magnetic crystals. They represent a model system for studying iron homeostasis and biomineralization in microorganisms. MTB sequester a large amount of iron in their crystals and have thus been proposed to significantly impact the iron biogeochemical cycle. Several studies proposed that MTB could also accumulate iron in a reservoir distinct from their crystals. Here, we present a chemical and magnetic methodology for quantifying the iron pools in the magnetotactic strain AMB-1. Results showed that most iron is not contained in crystals. We then adapted protocols for the fluorescent Fe(II) detection in bacteria and showed that iron could be detected outside crystals using fluorescence assays. This work suggests a more complex picture for iron homeostasis in MTB than previously thought. Because iron speciation controls its fate in the environment, our results also provide important insights into the geochemical impact of MTB.

## Abstract (precis)

Magnetotactic bacteria (MTB) produce iron-based intracellular magnetic crystals. They represent a model system for studying iron homeostasis and biomineralization in microorganisms. MTB sequester a large amount of iron in their crystals and have thus been proposed to significantly impact the iron biogeochemical cycle. Several studies proposed that MTB could also accumulate iron in a reservoir distinct from their crystals. Here, we present a chemical and magnetic methodology for quantifying the iron pools in the magnetotactic strain AMB-1. Results showed that most iron is not contained in crystals. We then adapted protocols for the fluorescent Fe(II) detection in bacteria and showed that iron could be detected outside crystals using fluorescence assays. This work suggests a more complex picture for iron homeostasis in MTB than previously thought. Because iron speciation controls its fate in the environment, our results also provide important insights into the geochemical impact of MTB.

## INTRODUCTION

Many living organisms transform inorganic molecules into crystalline structures in a process called biomineralization. Magnetotactic bacteria (MTB) represent an elegant example of such organisms. They incorporate dissolved iron from their environment and precipitate it as magnetite [Fe(II)Fe(III)_2_O_4_] or greigite [Fe(II)Fe(III)_2_S_4_] nanoparticles in organelles called magnetosomes (1). MTB are ubiquitous Gram-negative microorganisms in aquatic environments. They inhabit the oxic/anoxic transition zones in the water column or sediments where they thrive (2). In MTB, magnetosomes are aligned as chains inside the cell and provide the bacteria with a permanent magnetic dipole presumably for navigation purposes (1).

Tremendous work has been carried out to determine the biological and chemical reactions leading to magnetite synthesis in MTB (3). In the two best-studied, magnetite-forming strains *Magnetospirillum magneticum* AMB-1 and Magnetospirillum gryphiswaldense MSR-1, magnetosome formation is a genetically controlled process where (i) magnetosome vesicles are formed from invagination of the inner cell membrane, (ii) empty magnetosome vesicles are aligned as a chain inside the cell, (iii) iron is transported and concentrated into magnetosome for initiation of biomineralization, and (iv) crystal size and shape are precisely controlled in a species-specific manner. A set of ∼30 genes, located in a distinct portion of the genome called the magnetosome island (MAI), is required and sufficient for the stepwise formation of magnetosomes (4). Recently, iron isotope studies have provided an integrative model for iron uptake and precipitation as magnetite in the magnetotactic strain AMB-1 (5, 6). This model assumes that dissolved Fe(II) or Fe(III) species are incorporated into AMB-1 and stored in the cytoplasm and/or periplasm as Fe(III). This Fe(III) pool is then partially reduced into Fe(II) for trafficking to magnetosomes and oxidized for precipitation as magnetite (5). Direct mass spectrometry measurements of iron content and iron isotope composition in AMB-1 cells devoid of magnetosomes suggest that a large pool of iron, which could represent at least 50% of the total cellular iron, accumulates in reservoir(s) distinct from magnetite (5, 6). The abovementioned high-resolution mass spectrometry measurements of iron showed discrepancy with previous X-ray absorption analyses performed on both AMB-1 and MSR-1 strains, in which magnetite was the sole iron species detected at the end of the biomineralization process (7, 8). In these X-ray absorption studies, time course experiments were carried out in which AMB-1 or MSR-1 cells were cultivated without iron. When saturation of cell density was reached, iron was added to the growth medium to trigger magnetite biomineralization. These time-resolved spectroscopic characterizations of iron in AMB-1, MSR-1, and MS-1 identified several iron phases (e.g., ferrihydrite or ferrous iron) over magnetite biomineralization (7, 8). When biomineralization was complete, magnetite was the sole iron carrier observed in bacteria. Additional Mössbauer characterizations of iron phases in MS-1 and MSR-1 also found that magnetite was the main pool of iron in MTB (9, 10).

**FIG 1:**
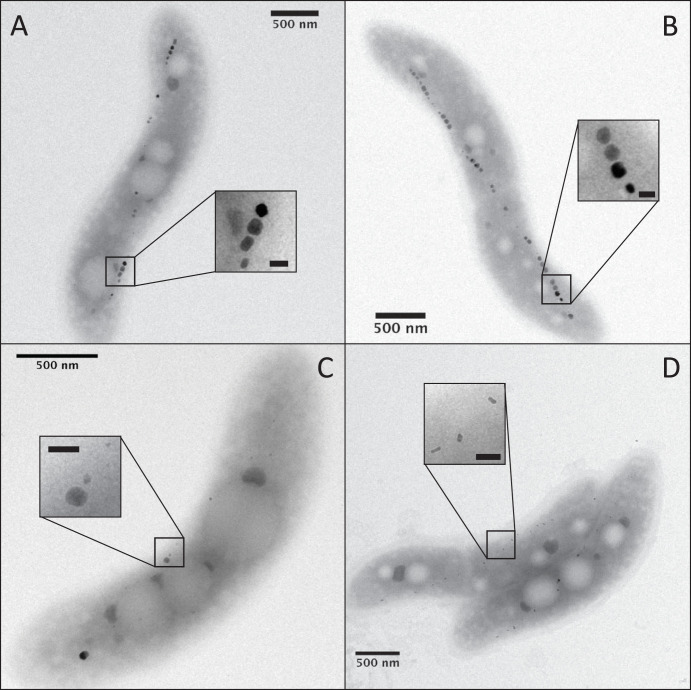
Transmission electron microscopy observations of wild-type AMB-1 cells cultivated for 3 days with initial iron concentrations in the growth medium of 30 (A) or 150 (B) μM and Δ*mamP* (C) and Δ*mamTΔ*R9 (referred to as Δ*mamT*) (D) AMB-1 strains cultivated with Fe(III)-citrate at 150 μM. The scale bars within the insets represent 50 nm.

In the present work, we address the discrepancy between the mass spectrometry and X-ray absorption experiments by determining the distribution of iron in AMB-1 cells. We grew AMB-1 with different iron concentrations and measured the mass of iron taken up by the bacteria using chemical assays. Cells were then recovered, and the mass of iron contained in magnetite was quantified from magnetic characterizations. From these experimental results, we found that magnetite represents ∼25% to ∼30% of the bulk cellular iron. Additional cultures of AMB-1 were grown to determine the total mass of iron contained in magnetite at the scale of a bacterial population. Simultaneous cell counting allowed us to estimate the mean mass of magnetite per cell. A comparison of these results with published single-cell quantification of bulk iron in AMB-1 supported mass balance estimations. Finally, we used a fluorescent reporter of iron to show that at least part of the noncrystalline iron is present as Fe(II) species within bacterial cells. To further investigate the link between magnetite formation and iron incorporation, mutant AMB-1 strains (Δ*mamP* and Δ*mamT* strains lacking the protein MamP and MamT, respectively) showing biomineralization defects were also analyzed following the same approaches (Fig. 1). Bacterial cultures of all strains were carried out in triplicates.

All data support the presence of a large pool of iron, at least partially reduced, distinct from magnetite in AMB-1 under our experimental conditions. These results raise important biochemical (i.e., iron homeostasis in MTB) and geochemical (i.e., impact of MTB on the iron biogeochemical cycle) questions that we address in the discussion of the manuscript.

## RESULTS

### Iron depletion and speciation in AMB-1 cultures.

**FIG 2:**
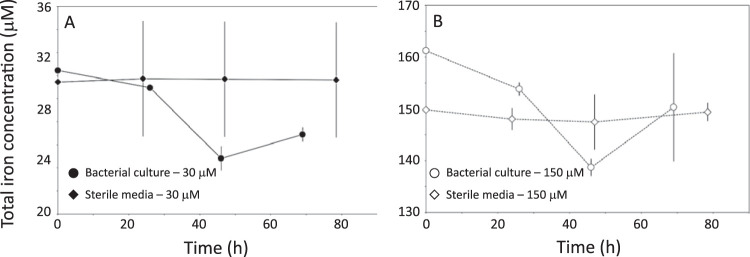
Total iron concentration in AMB-1 (circles) and sterile media (diamonds) provided with iron at 30 (A) or 150 (B) μM. Each point corresponds to the mean value of 3 replicates ± 1 SD. Note the different y axes.

**FIG 3:**
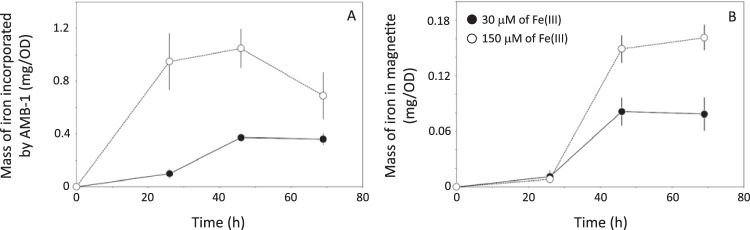
Mass of iron taken up by AMB-1 (A) and contained in magnetite during bacterial growth (B). All values are normalized to optical densities (ODs), which are proportional to the cell biomass. Thus, different cell densities cannot explain discrepancies in iron uptake. Each point corresponds to the mean value of three replicates ± 1 SD. Note the different y axes. Black circles and open symbols refer to cultures with initial iron concentrations of 30 and 150 μM, respectively.

**FIG 4:**
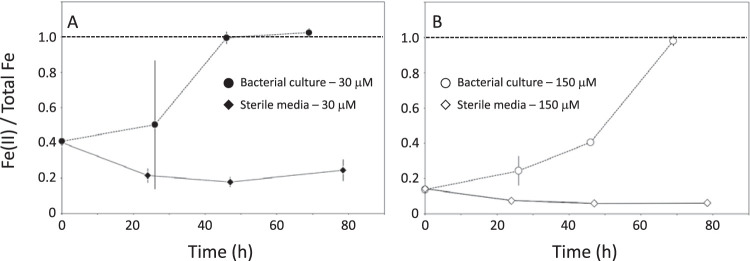
Iron speciation in AMB-1 (circles) and sterile media (diamonds) provided with iron at 30 (A) or 150 (B) μM. Each point corresponds to the mean value of 3 replicates ± 1 SD.

We first cultivated wild-type AMB-1 (Fig. 1) for 3 days at 2 iron concentrations (30 and 150 μM). Sterile media containing no bacteria were also prepared and used as a control condition. The iron concentration and oxidation state were monitored in AMB-1 cultures and sterile media using the ferrozine assay (see Materials and Methods). Iron concentration and oxidation state in the filtered sterile media remained constant over the 3 days of incubation, showing that all iron in the growth media can be analyzed by the ferrozine assay (Table 1; Fig. 2). Therefore, the decrease in iron concentration in the growth media is not due to precipitation of small iron phases excluded during filtration and can be attributed to iron uptake by AMB-1. Initial and final Fe(II), total iron concentration, and pH in wild-type AMB-1 growth media, as well as final optical densities at 400 nm (OD_400_s), are given in Table 2. Most of the bacterial iron uptake, normalized to biomass, occurred between 1 and 2 days of culture (Fig. 2). Iron depletion was 0.10 ± 0.04 and 0.95 ± 0.21 mg per unit of optical density (means ± standard deviations) after 26 hours of culture for an initial iron concentration of 30 and 150 μM, respectively, and 0.37 ± 0.03 and 1.05 ± 0.15 mg per unit of optical density after 46 hours of culture for an initial iron concentration of 30 and 150 μM, respectively (Fig. 3A). For an initial concentration of 30 μM, no further iron depletion occurred after 46 hours of culture. In contrast, the mass of iron depleted from the growth medium decreased to 0.69 ± 0.18 mg per unit of optical density in cultures provided with 150 μM iron. Iron speciation was also modified over bacterial growth. A total of 40% and 17% of the initial Fe(III) added to the growth media containing 30 and 150 μM iron, respectively, became immediately reduced after inoculation (Fig. 4). Remaining Fe(III) was then progressively reduced until complete reduction, which happened after 46 and 69 hours of culture for an initial iron concentration of 30 and 150 μM, respectively. Finally, the growth medium pH showed a similar increase between the two iron conditions, with final values of ∼7.5 (Table 2).

**TABLE 1:** Fe(II) and total Fe concentrations in initial and final sterile growth media

Time of culture (h) by concn	Vol of culture (ml)	Initial [Fe(II)] (μM)	Initial [Fe] (μM)	Final [Fe(II)] (μM)	Final [Fe] (μM)
[Fe] = 30 μM					
24	190	21.41	33.07	8.53	33.59
	190	18.97	31.50	6.61	30.28
	190	18.27	32.02	5.39	31.33
47	190	14.44	30.46	6.70	32.10
	190	12.18	32.20	5.29	31.22
	190	11.14	30.46	4.94	31.75
78.5	190	8.70	31.15	9.88	31.22
	190	5.22	30.98	6.88	32.28
	190	4.35	31.15	6.53	31.39
[Fe] = 150 μM					
24	190	33.07	151.58	11.83	146.53
	190	29.59	151.06	10.44	149.49
	190	30.63	150.71		
47	190	24.02	152.45	10.93	153.27
	190	22.28	139.57	8.64	146.39
	190	19.14	151.41	7.05	142.68
78.5	190	12.53	153.49	10.41	147.80
	190	11.66	149.49	9.70	149.03
	190	8.70	148.45	7.58	151.33

**TABLE 2:** Fe(II) and total Fe concentrations in the growth medium before and after AMB-1 cultures and final optical density and pH values of bacterial cultures

Time of culture (h) by concn	Vol of culture (ml)	Initial [Fe(II)] (μM)	Initial [Fe] (μM)	Final OD_400_ (AU^*a*^)	Final pH	Final [Fe(II)] (μM)	Final [Fe] (μM)
[Fe] = 30 μM							
26	185	16.87	31.36	0.067	6.95	7.29	30.94
	185	17.24	31.72	0.060	6.91	11.03	30.94
	185	17.05	31.72	0.083	7.01	28.45	30.94
46	185	14.12	32.46	0.209	7.26	24.99	24.30
	185	13.94	32.82	0.202	7.34	24.99	26.03
	185	14.85	31.72	0.214	7.26	24.12	24.12
69	185	8.80	33.19	0.212	7.42	28.12	27.42
	185	8.44	33.19	0.203	7.44	28.47	27.07
	185	7.52	33.74	0.204	7.42	26.20	26.20
[Fe] = 150 μM							
26	185	29.16	162.65	0.069	7.03	51.57	154.89
	185	26.96	157.15	0.067	6.94	35.21	152.40
	185	26.41	159.35	0.056	6.94	25.96	154.18
46	185	26.77	161.00	0.215	7.18	57.80	140.24
	185	23.29	158.25	0.215	7.28	54.15	138.85
	185	20.90	161.73	0.211	7.30	56.58	136.94
69	185	15.95	160.27	0.197	7.48	143.39	146.88
	175	15.95	172.00	0.202	7.50	154.57	162.08
	185	14.12	158.80	0.202	7.50	143.74	141.99

a AU, absorbance unit.

Iron uptake by the two mutant Δ*mamP* and Δ*mamT* strains was then measured following the same experimental procedure. Because mutant strains produce less magnetite than wild-type AMB-1, as shown by the electron microscope observations (Fig. 1), we cultivated them with iron at 150 μM to measure iron uptake more accurately. The highest iron uptake by wild-type AMB-1 was observed at ∼45 h of culture (see results). Accordingly, Δ*mamP* and Δ*mamT* strains were cultivated for ∼45 hours. Wild-type AMB-1 cultures were used as controls. The pH and optical density (OD) values were almost identical in all cultures of the three wild-type control and Δ*mamP* and Δ*mamT* AMB-1 strains (Table 3). Δ*mamP* cells showed limited iron incorporation normalized to the optical density, with an ∼4-fold decrease compared with the wild type (Table 3). Iron uptake by Δ*mamT* cells showed inconsistent values, with bulk iron concentration showing a slight increase during bacterial growth in two of the three replicates and a decrease in the third replicate. We considered these data to be inconclusive. Finally, the Fe(II)/total Fe ratios were slightly lower in Δ*mamP* (0.11 ± 0.02) and Δ*mamT* (0.09 ± 0.02) than that in wild-type AMB-1 (0.16 ± 0.03).

**TABLE 3:** Fe(II) and total Fe concentrations in the growth medium before and after mutant AMB-1 cultures, and final optical density and pH values of bacterial cultures^*a*^

Time of culture (h) by strain	Vol of culture (ml)	Initial [Fe(II)] (μM)	Initial [Fe] (μM)	Final OD_400_ (AU)	Final pH	Final [Fe(II)] (μM)	Final [Fe] (μM)
Wild type							
43	178	25.08	150.30	0.250	7.30	26.23	145.78
	173	22.85	155.69	0.252	7.24	25.23	150.59
	167	23.22	164.05	0.255	7.40	19.42	151.99
Δ*mamP*							
43	171	21.55	159.59	0.261	7.32	21.43	158.20
	174	19.32	161.27	0.252	7.35	18.23	161.00
	170	18.21	162.75	0.261	7.33	14.42	159.20
Δ*mamT*							
43	175	16.72	157.92	0.248	7.34	15.02	158.20
	177	15.42	153.23	0.280	7.35	15.62	148.29
	164	14.31	169.44	0.252	7.39	10.21	172.22

a Additional wild-type cultures were used as a control condition.

### Magnetic properties of AMB-1 cultures.

After chemical analyses, bacteria were recovered and transferred into sample holders for magnetic analyses. Hysteresis loops were measured on whole bacterial populations (see Fig. S1 in the supplemental material). Sample preparation was performed under anoxic conditions to prevent magnetite oxidation into maghemite [γ-Fe_2_O_3_]. The following three magnetic parameters were extracted from hysteresis loops: the remanent magnetization (M_rs_), the saturation magnetization (M_s_), and the coercivity (H_c_). M_s_ depends only on the mass of magnetic material for a given phase and is 92 emu (electromagnetic units) per gram of magnetite (11). Hysteresis measurements can detect and quantify all magnetic crystals in a given sample, even in the case of mixings of stable single-domain and small superparamagnetic particles observed in AMB-1 (see Fig. S2 and S3 in the supplemental material) (12–14). Iron phases in MTB have been extensively described. In AMB-1 and MSR-1, as well as in the closely related strain Magnetospirillum magnetotacticum MS-1, three iron species were found both from bulk measurements and observations at the atomic scale, namely, magnetite, ferrihydrite, and Fe^2+^ (7–10, 15). Ferrihydrite and Fe^2+^ are paramagnetic at room temperature and do not contribute to the M_s_ signal (16–18). The M_s_ values thus provide accurate estimates of the mass of iron in magnetite, which is a ferrimagnetic material. M_rs_ corresponds to the remanent sample magnetization measured under an external magnetic field of zero, after exposing the sample to a saturating external field. The M_rs_/M_s_ ratio depends on particle size and organization and typically ranges between 0.43 and 0.50 in AMB-1 (12, 13). Finally, H_c_ is the magnetic field strength required to reduce the magnetization of the sample to zero after fully magnetizing it. Thus, H_c_ represents the capability of a magnetic material to resist demagnetization. It depends on the particle size, its shape, its magnetization, and its magnetocrystalline anisotropy.

**FIG 5:**
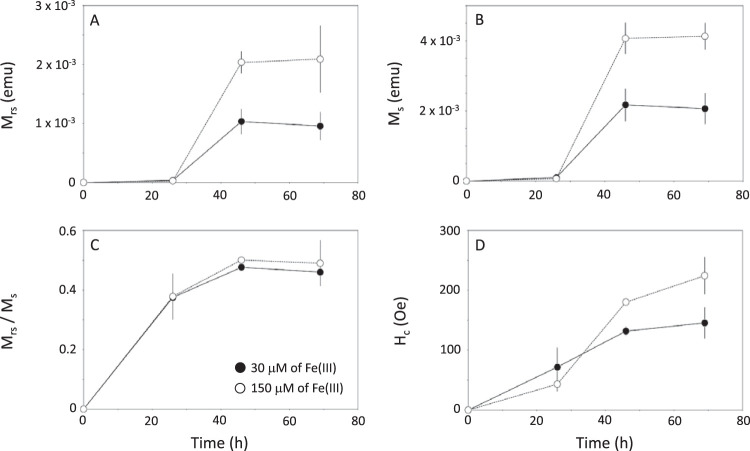
Remanent magnetization (M_rs_) (A), saturation magnetization (M_s_) (B), M_rs_/M_s_ ratios (C), and coercivity (H_c_) (D) for the studied AMB-1 cultures. Each point corresponds to the mean value of three replicates ± 1 SD. Black circles and open symbols refer to cultures with initial iron concentrations of 30 and 150 μM, respectively.

Magnetic parameters calculated from hysteresis loops (Fig. S1) are given in Table 4 and Fig. 5. After 26 hours of growth, remanent (M_rs_) and saturation (M_s_) magnetizations were similar between the two experimental conditions (Fig. 5A and B). Similar to iron uptake patterns, most magnetite was precipitated between 26 and 46 h of culturing under both conditions. No variation in M_rs_ and M_s_ was observed for a longer culture time, suggesting a complete magnetite biomineralization. Final M_rs_ was 9.57 × 10^−4^ ± 2.41 × 10^−4 ^emu and 2.09 × 10^−3^ ± 0.57 × 10^−3 ^emu for AMB-1 cultivated with iron at 30 and 150 μM, respectively. Final M_s_ values were 2.06 × 10^−3^ ± 0.44 × 10^−3^ and 4.13  × 10^−3^ ± 0.38 × 10^−3 ^emu for AMB-1 cultivated with iron at 30 and 150 μM, respectively. Knowing the magnetic moment of magnetite per unit of mass (92 emu/g), the M_s_ values were converted to a mass of iron contained in magnetite. Results are given in Fig. 3B. The maximum mass of iron in magnetite measured was 0.082 ± 0.015 and 0.15 ± 0.015 mg per unit of optical density in AMB-1 cultivated with 30 and 150 μM iron, respectively. From the remanent and saturation magnetization values, we calculated the M_rs_/M_s_ ratios. Almost identical values between the two experimental conditions were observed, namely, ∼0.38, ∼0.50, and ∼0.50 after 26, 46, and 69 hours of cultures, respectively (Fig. 5C). Coercivity showed a slightly different pattern, as it progressively increased with time (Fig. 5D). After 26 hours of growth, the coercivity of AMB-1 cultures was ∼50 Oe for both initial iron concentrations. At longer growth times, AMB-1 cultivated with 150 μM showed higher coercivities (180 ± 5 Oe and 224 ± 31 Oe for 46 and 69 hours of growth, respectively) than AMB-1 cultivated with 30 μM iron (132 ± 8 Oe and 146 ± 26 Oe for 46 and 69 hours of growth, respectively).

**TABLE 4:** Remanent magnetization, saturation magnetization, coercivity, and M_rs_/M_s_ ratios of wild-type AMB-1^*a*^

Time of culture (h) by concn	M_rs_ (emu)	M_s_ (emu)	H_c_ (Oe)	M_rs_/M_s_
[Fe] = 30 μM				
26	1.74 × 10^−5^	4.06 × 10^−5^	75.50	0.43
	2.55 × 10^−5^	7.88 × 10^−5^	37.02	0.32
	7.03 × 10^−5^	1.89 × 10^−4^	102.17	0.37
46	1.08 × 10^−3^	2.23 × 10^−3^	138.02	0.48
	8.00 × 10^−4^	1.68 × 10^−3^	123.28	0.48
	1.22 × 10^−3^	2.60 × 10^−3^	134.23	0.47
69	6.80 × 10^−4^	1.57 × 10^−3^	115.97	0.43
	1.07 × 10^−3^	2.24 × 10^−3^	156.12	0.48
	1.12 × 10^−3^	2.39 × 10^−3^	164.70	0.47
[Fe] = 150 μM				
26	5.19 × 10^−5^	1.12 × 10^−4^	56.81	0.46
	1.99 × 10^−5^	6.42 × 10^−5^	34.87	0.31
	1.15 × 10^−5^	3.18 × 10^−5^	37.50	0.36
46	2.23 × 10^−3^	4.52 × 10^−3^	186.19	0.49
	2.01 × 10^−3^	4.03 × 10^−3^	175.58	0.50
	1.86 × 10^−3^	3.64 × 10^−3^	179.27	0.51
69				
	1.69 × 10^−3^	3.89 × 10^−3^	202.27	0.43
	2.49 × 10^−3^	4.57 × 10^−3^	246.70	0.55

a M_rs_, remanent magnetization; M_s_, saturation magnetization; H_c_, coercivity; Oe, oersteds.

We measured the mass of ferrimagnetic material in mutant AMB-1 strains following the same approach. Jones and coworkers showed that nanoparticles produced in the *ΔmamP* and *ΔmamT* cells also correspond to magnetite (19). Therefore, the same saturation magnetization per unit of mass (92 emu/g) was used to calculate the mass of iron contained in magnetite. Mutant AMB-1 strains showed altered magnetic properties (Table 5). The remanent and saturation magnetizations in the Δ*mamP* strain were ∼1 order of magnitude lower than that of the wild type. Associated M_rs_/M_s_ ratios showed lower values in Δ*mamP* AMB-1 (0.34 ± 0.09) than that in the wild-type control (0.46 ± 0.05). In *ΔmamT* AMB-1, the saturation magnetization showed even lower values (∼2 orders of magnitude lower than the wild-type strain), while the remanent magnetization was ∼0. Accordingly, M_rs_/M_s_ ratios corresponding to magnetite produced by Δ*mamT* cells were also ∼0. Finally, coercivity showed an ∼3- to 4-fold decrease in the Δ*mamP* strain and was close to 0 in all Δ*mamT* mutant samples.

**TABLE 5:** Remanent magnetization, saturation magnetization, coercivity, and M_rs_/M_s_ ratios of whole mutant cells recovered after cultures^*a*^

Strain^*b*^	M_rs_ (emu)	M_s_ (emu)	H_c_ (Oe)	M_rs_/M_s_
Wild type	9.07 × 10^−4^	2.29 × 10^−3^	128.62	0.40
	8.06 × 10^−4^	1.64 × 10^−3^	274.89	0.49
	1.98 × 10^−3^	4.09 × 10^−3^	197.03	0.49
Δ*mamP*	6.92 × 10^−5^	2.09 × 10^−4^	64.10	0.33
	2.89 × 10^−5^	8.40 × 10^−5^	52.29	0.34
	1.94 × 10^−5^	5.56 × 10^−5^	44.71	0.35
Δ*mamT*	0	3.32 × 10^−5^	0	0
	3.58 × 10^−6^	3.85 × 10^−5^	0	0.09

a Additional wild-type cultures were used as a control condition. M_rs_, remanent magnetization; M_s_, saturation magnetization; H_c_, coercivity; Oe, oersteds.

b Time of culture was 43 h.

### Iron distribution in AMB-1 populations.

Having determined the mass of iron in the different bacterial pools (i.e., magnetite and the rest of the cell), we finally wanted to quantify the iron distribution in AMB-1. The mass of iron in the lysate (mass_lysate_; i.e., the fraction distinct from magnetite) was calculated from:

(1) $${\text{mass}}_{\text{lysate}}={\text{mass}}_{\text{cell}}-{\text{mass}}_{\text{magnetite}}$$

where mass_cell_ and mass_magnetite_ are the mass of iron contained in whole AMB-1 cells and in magnetite, respectively. mass_cell_ was calculated from chemical assays, and mass_magnetite_ was calculated from magnetic characterizations. The fraction of the total cellular iron contained in magnetite (F_magnetite_; in %) was calculated from the following:

(2) $${\text{F}}_{\text{magnetite}}=\frac{{\text{mass}}_{\text{cell}}-{\text{mass}}_{\text{lysate}}}{{\text{mass}}_{\text{cell}}}\times 100=\frac{{\text{mass}}_{\text{magnetite}}}{{\text{mass}}_{\text{cell}}}\times 100$$

where mass_lysate_ was calculated from Equation 1.

**FIG 6:**
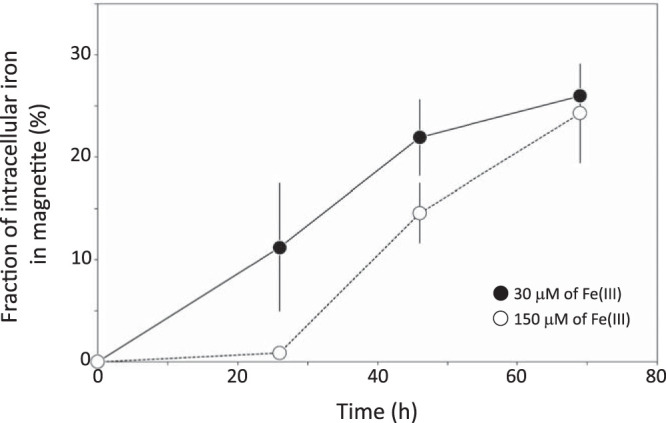
Relative fraction of the total cellular iron contained in magnetite. Each point corresponds to the mean value of three replicates ± 1 SD. Black circles and open symbols refer to cultures with initial iron concentrations of 30 and 150 μM, respectively.

Using results presented in Fig. 3A and B, we calculated the fraction of the total cellular iron contained in magnetite (F_magnetite_ in Equation 2) (Fig. 6). The masses of magnetite produced by wild-type AMB-1 cells cultivated at 30 and 150 μM were similar after 26 hours of growth, but iron uptake was 10 times higher under high iron conditions. Therefore, magnetite corresponded to 11.2% ± 6% of the total cellular iron at this time point under low iron conditions but only 0.9% ± 0.3% of the cellular iron under high iron conditions. In bacteria cultivated with 30 μM iron, the fraction of cellular iron in magnetite increased to 21.9% ± 4% and 26% ± 3% after 46 and 69 h of culture, respectively. Under the 150 μM iron experimental conditions, it increased up to 14.5% ± 3% and 24.3% ± 5% after 46 and 69 hours of culture, respectively (Fig. 6). We note that all cells from every sample observed under the electron microscope contained magnetite crystals. Therefore, our data cannot be explained by bacteria that accumulate iron without producing magnetite crystals. We manipulated and stored magnetite under anoxic conditions ([O_2_], <1 ppm) to prevent its oxidation into maghemite [γ-Fe(III)_2_O_3_] (20–22). The saturation magnetization of maghemite is 60 to 80 emu per gram (23). Even if all magnetite became fully oxidized, the mass of iron precipitated as crystals in wild-type AMB-1 would be at most 40% higher as in Fig. 3B, and the mineral fraction of AMB-1 would represent no more than ∼50% to 60% of the total cellular iron. In this case, our data would still support a significant pool of iron accumulating outside magnetosome crystals. Finally, we ensured that all iron fractions in AMB-1 cultures were recovered (see Materials and Methods). Thus, a loss of iron during sample extraction and preparation cannot explain our results. Our data demonstrate that magnetite does not represent the major iron reservoir in AMB-1 under our experimental conditions.

To further demonstrate that iron accumulates outside magnetite, we used additional wild-type AMB-1 cultures to assess the mean mass of magnetite per AMB-1 cell. Cell counting under a light microscope using a hemocytometer indicated an almost identical total number of cells for the two replicates, namely, 8.48 × 10^10^ ± 2.21 × 10^10^ and 8.39 × 10^10^ ± 2.26 × 10^10^. The total mass of magnetite produced in these cultures and calculated from magnetic measurements was 0.025 and 0.024 mg. This yields a mean mass of iron contained in magnetite per cell of 2.11 × 10^−7^ and 2.10 × 10^−7 ^ng for the two replicates, which corresponds to ∼21% of the bulk mass of iron measured in AMB-1 cells (24).

Additional wild-type cultures used as controls for experiments with the mutant strains showed similar results, with ∼31% ± 8% of the bulk cellular iron contained in magnetite. Magnetite in Δ*mamP* bacteria represented only 13% ± 12% of the total cellular iron. All Δ*mamP* cells also produced magnetite under our experimental conditions. Finally, the fraction of iron contained in Δ*mamT* magnetite could not be determined because of inconclusive data on iron incorporation into these mutant bacteria (see above).

### Subcellular localization and speciation of iron in AMB-1.

Iron distribution assessments demonstrated that AMB-1 cells contain a large pool of iron, distinct from magnetite. However, they did not provide physical and chemical information about this additional pool. To determine the subcellular localization and speciation of iron in AMB-1, we used the fluorescence resonance energy transfer (FRET) iron probe 1 (FIP-1), an activity-based probe that allows the detection of labile Fe(II) (25). FIP-1 is made of a green (fluorescein) and a red (cyanine) fluorophore linked by an Fe(II)-cleavable endoperoxide. In the native FIP-1 state, the fluorescence energy of the excited fluorescein is transferred to the cyanine through a FRET mechanism. In that case, only a red fluorescence signal can be observed. Upon reaction with labile Fe(II), the linker between the two fluorophores gets cleaved and a green fluorescence signal can be detected (25). Iron-induced FIP-1 cleavage can be detected from a measurement of green (green-dye excitation, green-dye emission) and FRET (green-dye emission, red-dye excitation) fluorescence intensity ratios. In that case, green fluorescence illustrates cleaved FIP-1 and FRET imaging indicates intact FIP-1. The green/FRET ratios typically increase by a factor of ∼2.5 upon iron-induced cleavage of FIP-1 (25). To further constrain the speciation and subcellular localization of iron distinct from magnetite in AMB-1, wild-type, Δ*mamP*, and Δ*mamT* cells were incubated with the FIP-1 probe and imaged via structured illumination microscopy. A mutant strain (ΔMAI) cultivated without iron and unable to form magnetosomes was used as a negative control (see supplemental text).

**FIG 7:**
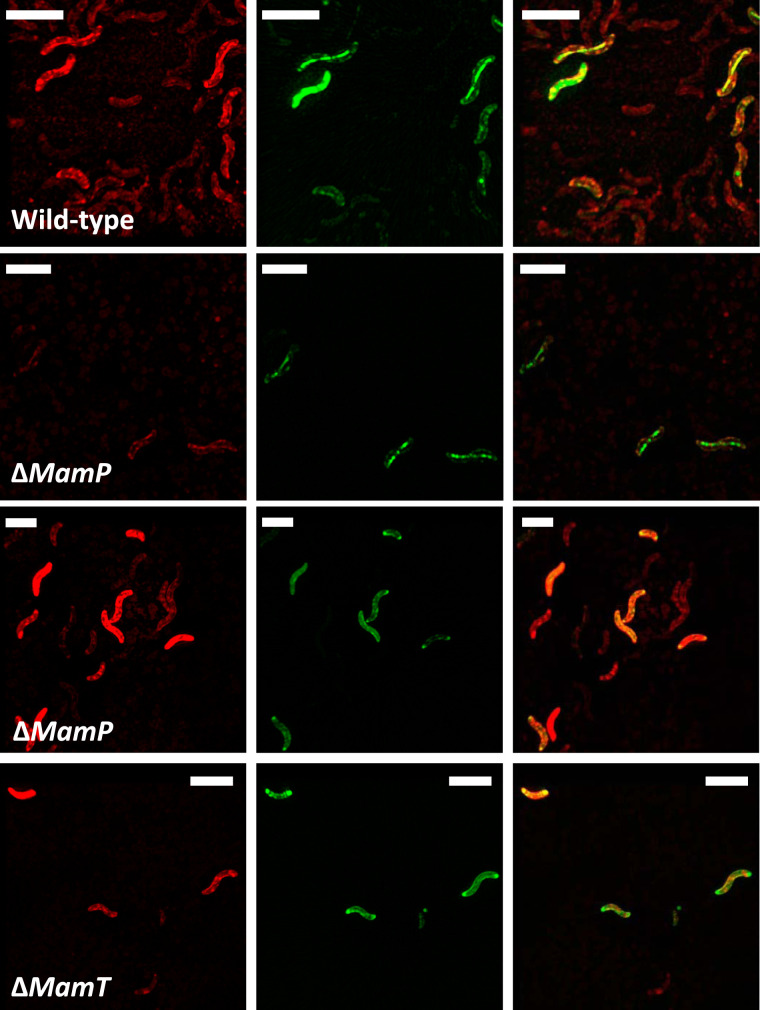
Red (left panels), green (center panels), and merged (right panels) fluorescence images obtained from superresolution microscopy of wild-type, Δ*mamP*, and Δ*mamT* AMB-1 incubated with FIP-1 for 180 min. The two Δ*mamP* panels show the two fluorescence patterns (diffuse and located to the magnetosome chains, as in wild-type) observed in the populations. Scale bars = 4 μm. Additional pictures are available in the supplemental material.

**FIG 8:**
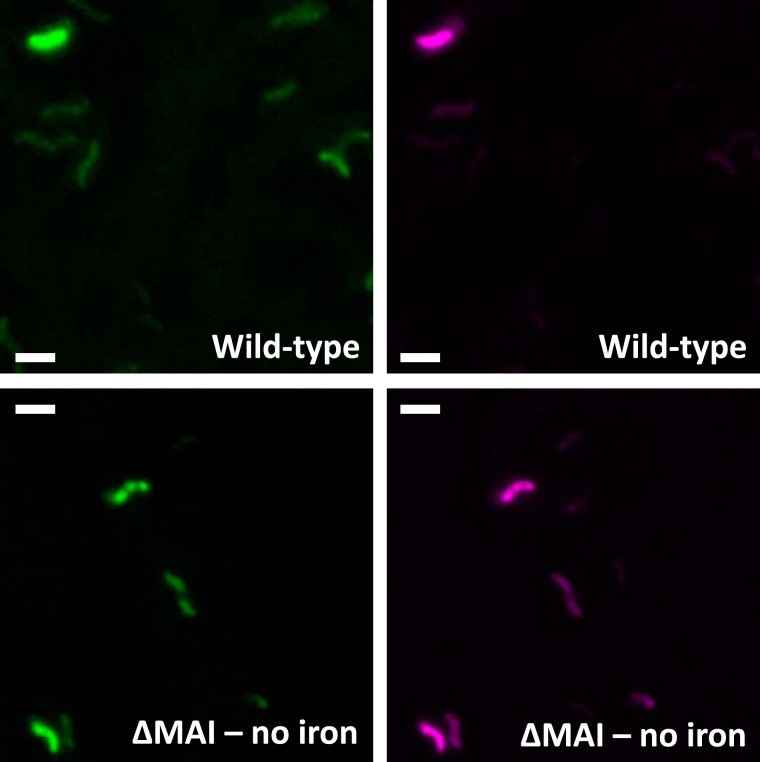
Green (left panels) and FRET (right panels) fluorescence images obtained from confocal microscope observations of wild-type AMB-1 cultivated with iron (top panels), and ΔMAI strain cultivated without iron (bottom panels). Scale bars = 3 μm.

**FIG 9:**
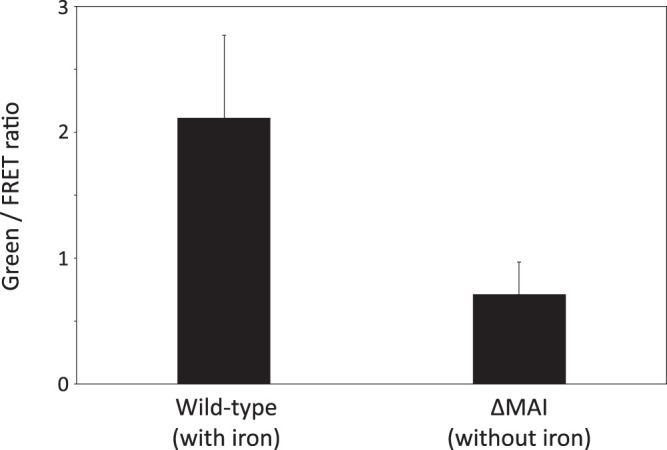
Mean green/FRET ratios measured with a confocal microscope in wild-type and ΔMAI AMB-1 cultivated with and without iron, respectively, and incubated with FIP-1 for 180 min.

A red fluorescence signal was observed in all samples incubated with FIP-1 for 90 min, indicating the uptake of FIP-1 (see Fig. S5 and S6 in the supplemental material). A very weak green signal was observed in ΔMAI bacteria (Fig. S6), suggesting a lower labile iron concentration in these mutant cells. This observation is in good agreement with the quantification of bulk iron in wild-type and ΔMAI bacteria (24). Both red and green fluorescence patterns showed intracellular heterogeneities, demonstrating that FIP-1 has been internalized into AMB-1. In wild-type bacteria, the green fluorescence signal was diffuse in the cytoplasm, although unstained spaces corresponding to PHB (poly-β-hydroxybutyrate; a carbon storage molecule) granules can be observed (Fig. S4 and S5). Green fluorescence signal also accumulated at the poles of the cell (Fig. S5). Such accumulation can be observed in dividing cells at the septum location (Fig. S7). Most of wild-type cells incubated with FIP-1 for 180 min also showed green fluorescence associated with the magnetosome chains (Fig. 7). To ensure that green fluorescence indicates iron-induced FIP-1 cleavage, we imaged wild-type and ΔMAI cells (∼100 cells for each strain) incubated with FIP-1 for 180 min using a confocal microscope (Fig. 8). The green/FRET fluorescence intensity ratios were quantified in both samples and showed a 3-fold decrease in the ΔMAI strain cultivated without iron (Fig. 9). These results are in good agreement with the work of Aron and coworkers (25). AMB-1 produces fragmented chains of magnetite, with magnetosome vesicles spreading along the cell’s long axis from pole to pole (26, 27). Unlike other *Magnetospirillum* strains such as MSR-1, apparent gaps between magnetite crystals can be observed from electron microscopy in AMB-1 (Fig. 1). These gaps correspond to empty magnetosome vesicles, containing no magnetite nanoparticles (26, 27). In our observations, the green fluorescence signals formed fragmented lines (i.e., similar to magnetite crystals) (Fig. 7; see also Fig. S8 in the supplemental material). In some rare cases, the fluorescence lines extended almost from poles to poles (i.e., similar to magnetosome vesicles). As mentioned above, all bacteria observed with electron microscopy contained magnetite nanoparticles. Therefore, fluorescence patterns showing continuous lines cannot indicate empty vesicles in cells making no magnetite. Δ*mamP* showed all of the fluorescence features that have been observed in the wild-type strain (Fig. 7; see also Fig. S9 in the supplemental material), whereas chains of magnetosomes could not be detected in Δ*mamT* AMB-1 using FIP-1 (Fig. 7; see also Fig. S10 in the supplemental material).

## DISCUSSION

Mass balance experiments identified a large amount of iron distinct from magnetite in AMB-1, representing ∼75% of the bulk cellular iron under our experimental conditions. These results suggest a more complex picture for iron cycling and homeostasis in MTB than previously thought, as intracellular iron needs to be handled by the cell to prevent toxic effects.

### Iron incorporation and distribution in AMB-1.

Monitoring iron concentration and oxidation state in AMB-1 growth medium demonstrated that initial Fe(III) became progressively reduced into Fe(II) (Fig. 4). Accumulation of Fe(II) could result from active reduction by AMB-1 or illustrate respiration reactions depleting oxygen in AMB-1 medium. Our experimental setup cannot rule out one of the two possibilities, but we note that iron isotopes identified Fe(III) reduction within AMB-1 cells and subsequent diffusion of intracellular Fe(II) to the growth medium (5).

Iron incorporation into wild-type AMB-1 was higher under high iron conditions. When normalized to optical density, which is proportional to the concentration of cells in culture, iron uptake by AMB-1 after 26 h of culture was ∼10-fold higher under the 150 μM experimental condition, than that under the 30 μM condition (Fig. 3). However, the mass of magnetite was similar under the two culture conditions (Fig. 3), indicating that the limiting step for biomineralization corresponds to magnetite precipitation and maturation, rather than iron uptake into the cell. Mass balance estimations were consistent in all wild-type cultures and indicated that ∼25% to ∼30% of the bulk cellular iron was contained in magnetite after 69 h of growth. The mean mass of iron contained in magnetite per cell (∼0.21 × 10^−6 ^ng), estimated from cell counting and magnetic quantification using a vibrating-sample magnetometer (VSM), corresponds to 21% of the bulk iron content in AMB-1 determined by single-cell mass spectrometry analyses under the same experimental conditions (24). The results are almost identical to the mass balance estimations and show that most iron is contained in reservoir(s) distinct from magnetite. Moreover, the combination of electron microscopy and mass spectrometry measurements for the quantification of iron content in AMB-1 evidenced a delay in magnetite formation, as iron was incorporated into bacteria (24). This observation further supports the accumulation of intracellular iron outside magnetite.

The limited iron incorporation into Δ*mamP* AMB-1 suggests that magnetite biomineralization regulates iron assimilation. Whether this regulation corresponds to a direct or indirect mechanism remains unclear. A likely hypothesis could be that the iron accumulation capacity of the cell’s fraction distinct from magnetite is limited. Once bacteria are fully loaded with iron, its sequestration into magnetite would be required for further assimilation. Such a model would imply a two-step process for magnetite biomineralization, in which iron is first stored in the noncrystalline fraction of the cell and then precipitated as magnetite. This idea is in good agreement with what has been proposed for iron cycling in AMB-1 and MSR-1 (5, 7, 8, 24). The lack of MamP in the mutant strain could hamper iron precipitation in magnetosomes and thus indirectly prevent further iron assimilation.

If 75% of intracellular iron in AMB-1 is not stored in magnetite, the pool distinct from magnetite should represent ∼0.75 × 10^−6 ^ng of iron per cell (24). This mass is estimated to be 10- to 100-fold higher than the mass of iron in Escherichia coli cells (28). The iron content in the ΔMAI AMB-1 strain, unable to form magnetosomes, was also estimated to be 5- to 10-fold higher than that in E. coli cells (24). An excess of free iron in the intracellular medium is toxic for cells (28), which suggests efficient iron storage and detoxifying pathways in AMB-1. They could include ferritins, bacterioferritins, and Dps proteins (15, 28, 29). Dps and bacterioferritins have recently been shown to protect MSR-1 from oxidative stress (15), and phases corresponding to ferritin-like structures have been evidenced in AMB-1, MSR-1, and MS-1 strains using spectroscopic methodologies (7–10, 15, 29). Further iron toxicity assays in MTB and mutant strains lacking some of these iron-storing proteins will help to better understand the capacity of MTB to tolerate such high intracellular iron concentrations.

### Comparison with previous work.

Previous research reported iron uptake by AMB-1 similar to our findings. Komeili and coworkers observed an iron incorporation of 0.03 mg per OD (400 nm) unit after 25 h of culture (30). In the present contribution, we observed an incorporation of 0.1 mg per OD (400 nm) unit. The mean size of magnetite crystals and the number of nanoparticles per cell were close to those of published results (31). Still, we note that Jones et al. (19) reported a higher magnetite size corresponding to a 2-fold increased volume. Overall, a literature survey showed that our results are in the range of published works. Even considering an increase of magnetite size similar to the work of Jones and coworkers, our results would still support a large pool of iron distinct from magnetite. The variations in iron uptake and magnetite size in AMB-1 likely illustrate distinct culture conditions.

Our results clearly showed discrepancy with previous characterizations of iron species in the two magnetotactic strains AMB-1 and MSR-1 using X-ray absorption methodologies (7, 8). Since additional iron was detected by mass spectrometry and the protocol described in the present research, such a discrepancy could suggest that X-ray absorption is not suitable for the detection of iron that is not contained in magnetite or ferrihydrite. However, the significant fraction of iron we identified in the iron pool distinct from magnetite suggests that the discrepancy arises from different experimental protocols. Moreover, recent work on MSR-1 also suggested that iron can be contained outside magnetite in this strain (32). Iron-starving conditions can impact the iron cycling and homeostasis in MTB, as low-iron conditions have been shown to induce overexpression of iron acquisition systems in AMB-1 and MSR-1 (33, 34). They might optimize the transfer of incorporated iron to magnetosomes for magnetite precipitation. Further X-ray absorption analyses with iron-starved bacteria and cells grown under standard conditions (i.e., as in the present work) will be needed to confirm this hypothesis.

### The magnetic properties of AMB-1 cultures illustrate defects in magnetite biomineralization.

In addition to the mass of magnetite produced in AMB-1 cultures, magnetic characterizations of bacterial samples provided important insights on the nanoparticle size and organization. AMB-1 produces stable single-domain magnetite nanoparticles (13). The M_rs_/M_s_ ratios corresponding to wild-type, mature AMB-1 magnetite typically range between 0.43 and 0.50 (12, 13). Smaller magnetite particles with crystal dimensions below 30 nm (for a width/length ratio of 0.2 or higher) are not magnetically stable at room temperature and fall within the superparamagnetic domain (35). Their remanence magnetization is thus 0 at room temperature, but their saturation magnetization remains unchanged for a given mass of magnetite. Very small superparamagnetic particles (<10 nm in length) would not reach complete saturation under the maximum external field we used (4,000 Oe) at room temperature (36). These particles represent less than 5% of the magnetite crystals observed under electron microscopy (Fig. S2). The underestimation of the saturation magnetization of these nanoparticles would be around 20% (36), meaning that the underestimation of the mass of magnetite would be below 1% and thus negligible. A mixing of stable single-domain and superparamagnetic particles would lead to lower M_rs_/M_s_ ratios (12). M_rs_/M_s_ ratios of ∼0.38 observed after 26 h of growth in wild-type cultures could thus reflect a mixing of mature and newly formed particles. Even though iron uptake was ∼10-fold higher under high-iron conditions (Fig. 3), M_rs_/M_s_ ratios in the 2 iron conditions were identical regardless of the initial iron concentration in the growth medium. As mentioned above, this suggests magnetite precipitation and growth as the limiting step for biomineralization in AMB-1. For longer culture times, M_rs_/M_s_ ratios in wild-type AMB-1 were consistent with values ranging between 0.46 and 0.49 typical of AMB-1 magnetite (13). Δ*mamP* and Δ*mamT* AMB-1 also showed decreased M_rs_/M_s_ ratios compared with the expected ∼0.45 value. M_rs_/M_s_ ratios in Δ*mamP* cultures of 0.34 are consistent with a mixing of stable single-domain magnetite and smaller superparamagnetic particles, as confirmed by electron microscopy observations (Fig. 1). As mentioned above, very small superparamagnetic particles would not reach saturation at room temperature, leading to an underestimation of the mass of magnetite of ∼20%. In that case, our results would still support a large pool of iron distinct from magnetite in the Δ*mamP* strain. Contrastingly, Δ*mamT* showed M_rs_ and M_rs_/M_s_ ratios of zero, which are both consistent with superparamagnetic particles produced in this strain. Electron microscopy demonstrated that Δ*mamT* bacteria produced ∼10- to 20-nm-long nanoparticles, which is in good agreement with the magnetic analyses (35). However, a 100-fold decrease of M_s_ in Δ*mamT* AMB-1 as we observed (Table 5) is not expected from estimation of the magnetite volume in wild-type and Δ*mamT* strains (Fig. S2 and S3). This discrepancy could be explained by the presence of crystal phases distinct from magnetite, such as hematite (supplemental material).

Finally, coercivity in wild-type cultures increased with time under both iron conditions. Coercivity in stable single-domain particles, such as those produced by MTB (13), depends on the particle size and shape, as well as the magnetocrystalline anisotropy which is unlikely to change. Consistent H_c_ values between the two iron conditions after 26 h of culture suggest a similar size and shape for magnetite crystals, which is in good agreement with iron uptake patterns, remanent magnetization, and saturation magnetization (see above). For longer times of culture, AMB-1 cultivated with iron at 150 μM showed higher coercivity. Since M_rs_ is unchanged between these two cultures, this requires that the particles are larger or a different shape under high iron conditions. To test this hypothesis, we measured the magnetite length and width distributions under both experimental conditions. Results are given in Fig. S2 and S3 and show that the shape (length/width ratio) is almost identical under both experimental conditions. However, bigger magnetite crystals were produced by AMB-1 cultivated with iron at 150 μM (mean length of 38.46 nm) than those under the 30 μM condition (mean length of 32.03 nm), suggesting that increased coercivity results from bigger particles under high iron conditions. Lower coercivities in the mutant strains can also be explained by the presence of small superparamagnetic particles.

### Localizing iron in AMB-1 cells.

Two green fluorescence patterns were observed in AMB-1 cultures, namely, a diffuse signal across the cell and a signal that concentrated on the magnetosome chain. FIP-1 is made of the following two dyes: lipophobic fluorescein and lipophilic cyanine (37). It is worth mentioning that magnetosome chain-like fluorescence patterns cannot be generated by lipophilic artifacts, as only the green lipophobic dye (fluorescein) stained magnetosome chains. Furthermore, the lipophobic and lipophilic natures of fluorescein and cyanine, respectively, can explain holes in green images observed at the location of PHB granules (Fig. S5 and S7). Red fluorescence seemed to accumulate at these locations, thus generating mutually excluding green and red fluorescence patterns (merged panels in Fig. S5 and S7). This finding further demonstrates FIP-1 cleavage in AMB-1 cells, as such fluorescence patterns would be difficult to explain with intact probes. The observation of magnetosome chains using FIP-1 shows that Fe(II) is addressed to magnetosomes during biomineralization. There is a possibility that FIP-1 indicates poorly crystalline Fe(II) at the magnetite surface, but our observations are best explained by Fe(II) being contained either in magnetite-containing or magnetite-free magnetosome vesicles (see below). It is unclear whether this Fe(II) would be contained inside magnetosomes, within the magnetosome membrane, or at the magnetosome surface. From high-resolution electron microscope analyses, Werckmann and collaborators proposed that iron could accumulate in the magnetosome membrane before its precipitation as magnetite (38). Our observations are in line with these results and indicate that iron in the magnetosome membrane would at least be composed of Fe(II) species. Genes encoding Fe(II) transporters have been found in the magnetosome gene island and could transport Fe(II) across the magnetosome membrane for magnetite formation (33, 39, 40). Finally, AMB-1 cells showed heterogeneous fluorescence intensities. We have already shown that the mass of bulk intracellular iron in AMB-1 showed more than 10-fold variations between different cells under similar initial iron conditions (24). The number of magnetite crystals per AMB-1 cell was 18 ± 5 (Table S2). Therefore, the variation of bulk iron content in AMB-1 cannot be explained by various mass of magnetite but rather illustrates the additional pool of iron we identified. Such an observation is in good agreement with the variability in fluorescence intensity, as only labile Fe(II) can cleave FIP-1 (25).

Identical fluorescence patterns were observed in Δ*mamP* AMB-1 but not in the Δ*mamT* strain. The chain-like localization pattern in Δ*mamP* suggests that FIP-1 does not bind to magnetite since this mutant strain produces only a few crystals per cell. It also indicates that Fe(II) is delivered to magnetosomes in the AMB-1 cells lacking MamP and suggests that magnetite-free magnetosomes can be stained by FIP-1.

Another notable observation is an accumulation of fluorescence at the poles of AMB-1 cells showing a diffuse green signal. Whether such an accumulation illustrates true biological mechanisms (flagellar apparatus, chemotaxis receptors, nitrate reductase complex, siderophore-specific multienzymatic complexes called siderosomes, and cell division) remains speculative, and additional work will be necessary to determine the significance of these observations (41–45).

Last, it is important to note that FIP-1 does not react with Fe(II) bound tightly to proteins as well as Fe(III) (25). It is likely that additional iron species distinct from magnetite are contained in AMB-1 cells, which include iron associated with heme domains (19, 46) or iron contained in storage proteins, such as ferritins (10, 15).

### Implications for Earth sciences.

It has been hypothesized that MTB deplete their environment in bioavailable iron by sequestering dissolved species into magnetite (47). Once the cell dies, MTB magnetite crystals can be trapped into sedimentary rocks, which effectively removes iron from the dissolved pool (2, 48). MTB could thus prevent other living organisms from accessing an available source of iron. Some parameters are missing to accurately quantify the impact of MTB on the iron biogeochemical cycle. One of them is the speciation of iron in MTB, which controls its solubility. Our data demonstrated that most iron in MTB exists as soluble species [i.e., Fe(II) and soluble Fe(III) organic compounds] rather than magnetite. Iron sequestration in environmental MTB might thus be more limited than that previously proposed (24). The additional pool of iron we identified could also have implications for environmental magnetism but only if such iron could precipitate as magnetic phases (e.g., magnetite, hematite, and pyrite). In this case, the fate of iron in the environment would depend on the physicochemical conditions at the location of MTB iron release. However, the discrepancy between the present work and the former X-ray absorption characterizations of iron in MTB (7, 8) raises questions about the environmental significance of our findings. Environmental MTB populations could experience various iron conditions and transition from iron-starving to iron-rich conditions, similar to X-ray absorption experiments (7, 8). In this case, most intracellular iron could be contained in magnetite, with a limited soluble iron pool. Additional work constraining iron speciation in MTB that experience transitioning iron conditions, as well as in bacterial populations sampled from the environment, will be useful to further address the impact of MTB on the iron biogeochemical cycle.

## MATERIALS AND METHODS

### Cultivation of wild-type and mutant AMB-1 strains.

*Magnetospirillum magneticum* AMB-1 (ATCC 700264) was cultivated in 200-ml bottles. The detailed composition of AMB-1 growth medium is given by Komeili and coworkers (30). The sole iron source provided to AMB-1 cultures corresponded to Fe(III)-citrate, which was added to the growth media from an Fe(III)-citrate solution prepared by mixing Fe(III)Cl_3_ (6 mM) and citric acid (12 mM) powders (Sigma-Aldrich) in Milli-Q water. The pH of the Fe(III)-citrate solution was set at 6.9 (i.e., same as AMB-1 growth medium) using NaOH. The initial Fe(III) concentration in AMB-1 growth medium was either 30 μM (i.e., standard concentration used in the ATCC medium) or 150 μM (i.e., the concentration used for isotope experiments which was hypothesized to generate more unmineralized iron in AMB-1). The concentration of citrate and the volume of cultures were kept constant in all experiments by adding an iron-free citrate solution (12 mM; pH 6.9) to AMB-1 cultures under low iron conditions (30 μM). AMB-1 was cultivated in a glove box with controlled atmosphere (90% N_2_; 10% O_2_) at 30°C for 3 days. Each day, one bottle for each experimental condition was recovered for chemical and magnetic characterizations (see below). All measurements were carried out in triplicates (total of 18 bottles: 2 iron conditions, 3 time points, and 3 replicates for each condition).

The following two AMB-1 mutant strains were selected for additional experiments: the *ΔmamP* and Δ*mamT* strains lacking the genes encoding the MamP and MamT proteins, respectively (4). MamP and MamT are magnetochrome proteins, a class of c-type cytochromes specific to MTB, which can bind iron via their heme domains (46). Magnetochromes have been proposed to regulate the iron oxidation state in MTB (46). The two mutant strains show biomineralization defects, which enable us to investigate the link between magnetite formation and iron uptake. The *ΔmamP* strain produces only a few crystals per cell resembling those produced by wild-type AMB-1, as well as a few additional small crystals (Fig. 1A to C). Δ*mamT* bacteria synthesize many small, elongated crystals (Fig. 1D). The *ΔmamP* and *ΔmamT* strains have already been produced by our group (4). In AMB-1, the *mamT* gene is located in the *mamAB* gene clusters of the MAI (termed R5 region in our previous work) downstream of three genes, namely, *mamQ*, *mamR*, and *mamB* (4). These three genes are perfectly duplicated in the R9 region of the MAI, downstream of *mamT*. To avoid recombination between regions R5 and R9, the region R9 was deleted from the *ΔmamT* strain. Therefore, bacteria used in this study correspond to the *ΔmamTΔ*R9 strain and are referred to as Δ*mamT*. We ensured that *ΔmamT* and *ΔmamTΔ*R9 strains show similar biomineralization defects and both can be complemented with *mamT* expressed from a plasmid (19). Because the mutant strains produce less magnetite than wild-type AMB-1, as shown by the electron microscope observations (Fig. 1), we cultivated them with Fe(III)-citrate at 150 μM to measure iron uptake more accurately. The highest iron uptake by wild-type AMB-1 was observed at ∼45 h of culture (see Results). Accordingly, *ΔmamP* and *ΔmamT* strains were cultivated for ∼45 hours in triplicates in 200-ml bottles (total of 9 bottles: 3 replicates each for *ΔmamP* and *ΔmamT* and 3 replicates for wild-type bacteria used as a control).

### Transmission electron microscopy.

Bacteria were deposited on copper grids coated with a Formvar film and observed with a transmission electron microscope (FEI Tecnai 12) operating at 120 kV. From electron microscopy observations, the length and width of magnetite nanoparticles produced by wild-type AMB-1 cultivated for 3 days with Fe(III) at either 30 or 150 μM were measured using the ImageJ software. The sizes of mutant AMB-1 crystals were also measured using Image J.

### Chemical measurements.

Bacterial iron uptake was quantified by measuring iron concentration in AMB-1 cultures at initial (immediately after inoculation) and final (at the end of the bacterial culture) stages using the ferrozine assay (49). Ferrozine forms a purple-colored complex with Fe(II), which can be determined spectrophotometrically. Total iron is then determined by total reduction of iron in the sample with hydroxylamine hydrochloride and subsequent reaction with ferrozine. Concentration of Fe(III) is calculated as the difference of total iron and Fe(II). For each condition, pH and optical density at 400 nm (OD_400_) were measured. Then, 1 ml of culture was sampled and filtered (0.22-μm pore size; Acrodisc syringe filters, polyethersulfone) at the initial and final stages. The Fe(II) and total iron concentrations were measured using the ferrozine assay. The mass of iron taken up by AMB-1 was calculated from iron depletion in each culture.

To demonstrate the reliability of the ferrozine assay for measuring iron depletion in AMB-1 cultures, we also prepared sterile growth media provided with Fe(III)-citrate at 30 or 150 μM in 200-ml bottles. One milliliter of growth media was sampled and filtered (0.22-μm pore size; Acrodisc syringe filters, polyethersulfone) after iron addition. Iron concentration and speciation was measured with the ferrozine assay as described above. Sterile bottles were incubated for 1, 2, or 3 days at 30°C in the glove box (90% N_2_, 10% O_2_). At the end of the experiment, 1 ml of growth media was recovered and filtered and the iron concentration and speciation were measured using the ferrozine assay. Three replicates per condition were prepared (18 samples total, as for wild-type bacterial cultures).

### Magnetic characterizations.

After chemical analyses, whole growth media were recovered and centrifuged (8,000 rpm; 10 min). Supernatants were discarded, and bacterial pellets corresponding to the entire bacterial populations were dried in an anoxic chamber (98% N_2_, 2% H_2_, O_2_ of <1 ppm) at room temperature to prevent magnetite oxidation. We have already demonstrated that no significant fraction of iron is adsorbed on AMB-1 cell surfaces (5). Virtually all iron contained in bacterial pellets thus corresponds to intracellular iron. Once dried, whole bacterial pellets were transferred into sample holders inside the anoxic chamber for subsequent magnetic characterizations. Samples were kept in anoxic conditions until magnetic analyses were performed. Hysteresis loops of magnetization versus applied magnetic field were measured using a vibrating-sample magnetometer (LakeShore VSM 7410) at room temperature. An integration time of 10 s per point was used.

### Iron mass balance.

To demonstrate the validity of our protocol and the accuracy of our measurements, we ensured that all iron fractions were recovered and that no iron was lost during sample extraction and preparation. Additional wild-type AMB-1 cultures were carried out in 200-ml bottles for 3 days. One milliliters of growth medium was sampled and filtered before and after cultures, and iron concentration was measured using the ferrozine assay as described above. Cells were recovered by centrifugation (8,000 rpm; 10 min). The supernatant was discarded, and bacterial pellets were suspended in 100 μl of phosphate-buffered saline (PBS). Cells were washed three times in PBS and stored for subsequent measurements of total cellular iron mass (m_cell_) using single-cell inductively coupled plasma-mass spectrometry following a protocol previously published (24). Before mass spectrometry measurements, the PBS solution containing the bacteria was filtered to measure the potential mass of iron that leaked outside the cells using high-resolution inductively coupled plasma-mass spectrometry (m_leaked_) (24). Iron recovery was assessed from the following mass balance equation:

(3) $$m_{\text{initial medium}}=m_{\text{residual medium}}+m_{\text{cell}}+m_{\text{leaked}}$$

where m_initial medium_ and m_residual medium_ are the mass of iron in the growth medium before and after AMB-1 cultures, respectively. m_leaked_ represented ∼0.5% of m_cell_ or less (Table 1). Mass balance estimations showed that iron recovery during sample preparation was ranging between 96% and 100% (see Table S1 in the supplemental material), demonstrating the validity of our protocol. Therefore, a loss of iron pools such as magnetite could not explain our results.

### Cell counting.

To further demonstrate that wild-type AMB-1 incorporates more iron than needed to make its magnetite crystals, we chose an alternative approach to estimate the mean mass of magnetite per cell and to compare these results with available data on single-cell bulk iron content in AMB-1. Additional wild-type AMB-1 cultures (two replicates) were grown with Fe(III)-citrate at 150 μM. For each culture, the entire AMB-1 population was recovered with centrifugation, and the total mass of magnetite in a given population was determined from magnetic measurements as described above. The number of cells in the same populations was then calculated from direct cell counting using a hemocytometer under a light microscope. Fifty counts were done for each AMB-1 culture. Finally, the mean mass of iron per cell was calculated from the total number of cells and the total mass of magnetite in each culture.

### Detection and mapping of labile Fe(II) in AMB-1 using the FIP-1 fluorescent probe.

The sensing mechanism for FIP-1 is inspired by antimalarial natural products and related therapeutics (50–53). This reagent has been developed for use in mammalian cells and expanded in bioluminescent versions for mouse imaging (54). We adapted the use of FIP-1 (25) for detection of Fe(II) in AMB-1. Wild-type and mutant AMB-1 strains were cultivated in 10-ml glass tubes until end of the exponential phase/beginning of the stationary phase. Ten milliliters of growth medium was centrifuged, the supernatant was discarded, and cells were resuspended in 500 μl of PBS buffer. To ensure that all iron from the growth medium was removed, cells were centrifuged and washed in fresh PBS buffer three times. Finally, the three bacterial strains were mixed with a PBS solution containing EDTA (5 mM; pH 6.9) for 10 min, centrifuged, and suspended in the FIP-1 solution (i.e., FIP-1 at 1 mM in Hanks’ balanced salt solution) for 180 min at 30°C in the glove box (90% N_2_; 10% O_2_). All bacterial samples were observed by structured illumination microscopy with a Carl Zeiss Elyra PS.1 superresolution fluorescence microscope, using red (excitation wavelength of 561 nm, emission wavelength of 570 to 620 nm) and green (excitation wavelength of 488 nm, emission wavelength of 495 to 550 nm) laser lines for the detection of the native and cleaved probe, respectively. Green (excitation wavelength of 488 nm, emission wavelength of 495 to 550 nm) and FRET (excitation wavelength of 488 nm, emission wavelength of 570 to 620 nm) fluorescence intensity in wild-type and ΔMAI bacteria incubated with FIP-1 for 180 min were measured with a Carl Zeiss LSM 710 confocal microscope. Images were processed with the ImageJ software.

## Supplementary Material

Supplemental file 1
