# Supplementary material for: Magnetotactic Bacteria Accumulate a Large Pool of Iron Distinct from Their Magnetite Crystals
Source: Appl Environ Microbiol. 2020 Oct 28;86(22):e01278-20. doi: 10.1128/AEM.01278-20 (PMC7642088; doi:10.1128/AEM.01278-20)
Supplement: Supplemental file 1 [file AEM.01278-20-s0001.pdf]

Supplementary information for

Magnetotactic bacteria accumulate a large pool of iron distinct from their magnetite crystals

Matthieu Amor[1]*, Alejandro Ceballos[2], Juan Wan[1], Christian P. Simon[3], Allegra T. Aron[4,5,6],

Christopher J. Chang[4,7], Frances Hellman[2,3,8], Arash Komeili[1,7]

Corresponding authors: matthieu.amor@cea.fr (MA); komeili@berkeley.edu (AK)

Supplementary text

Incubation of a mutant AMB-1 strain unable to form magnetosomes with FIP-1

An AMB-1 mutant unable to form magnetosomes from which the entire MAI has been deleted (ΔMAI strain) was used as a negative control for FIP-1 experiments. ΔMAI cells were cultivated as described above in the absence of an iron source. To ensure that no iron contamination from the glassware occurs, tubes containing ΔMAI cells were soaked with an oxalic acid solution (concentration of 4.25 g per liter) overnight. After incubation with FIP-1, bacteria were centrifuged and suspended in fresh PBS to remove FIP-1 probes in the external solution. Wild-type and ΔMAI AMB-1 were incubated with FIP-1 for 90 min, and observed by Structured Illumination Microscopy with a Carl Zeiss Elyra PS.1 Super Resolution fluorescence microscope, as described in the main text. Results are shown in Figs. S4 and S5.

Magnetite size and shape factor

The length of magnetite crystals produced by wild-type AMB-1 was measured from electron microscopy images using the ImageJ software. Results are given in Fig. S2, and show longer crystals when AMB-1 is cultivated under higher iron conditions. The nanoparticle shape anisotropy was then quantified by measuring the shape factor (s) defined as:

$$s = \frac{width}{length} \qquad \text{(Eq. S1)}$$

Assuming a consistent shape factor, a linear relationship exists between the width and the length. Results are given in Fig. S3, and show a rough linear relationship between magnetite width and length, with correlation coefficients of 0.86 and 0.89 for iron concentration

conditions of 30 and 150 μM, respectively. Shape factors showed similar values (0.80 and 0.82) almost similar to what has been measured in AMB-1 before (1).

Saturation magnetization in mutant strains

The saturation magnetization measured in Δ*mamT* samples showed a 100-fold decrease compared to the wild-type strain (Table 5). From measurement of (*i*) the crystal size, (*ii*) the number of crystals per cell, and (*iii*) the shape factor (width/length ratio) in Δ*mamT* and wild-type strains (Table S2), only a ~30-fold decrease of magnetite volume and associated saturation magnetization can be expected. The detection limit of the VSM we used for the magnetic characterizations is 300 times lower than the magnetization in Δ*mamT* samples. Even though 2D projections of 3D objects can generate biases, the lower saturation magnetization than expected in Δ*mamT* AMB-1 could be explained by crystal phases distinct from magnetite and showing lower saturation magnetization. One example of such phases include hematite, which has a saturation magnetization of 0.3 emu/g (2). From single-crystal characterization, Jones and co-workers identified only magnetite crystals in Δ*mamT* AMB-1 (3). Therefore, it is possible that some other crystal phases were missed. Dedicated experimental work and bulk characterizations of iron phases in Δ*mamT* AMB-1 will be needed to address this question.

Crystal phases in Δ*mamP* AMB-1 were characterized by Jones and co-workers following the same single-crystal approach (3). Therefore, additional crystal phases than magnetite might as well be contained in this mutant strain. However, the decrease in saturation magnetization of Δ*mamP* compared to the wild-type strain expected from electron microscopy (~10-fold, see Table S2) showed a much lower discrepancy than the one we measured (~20-fold, see Table 5). In this case, the discrepancy between expected and measured saturation magnetization could also be explained from biases generated by 2D projection of 3D objects. Moreover,

magnetite porosity in MTB has never been characterized. The density of magnetite could also show variations between the several AMB-1 strains we tested.

Supplementary references

1. Li J, Pan Y, Chen G, Liu Q, Tian L, Lin W. 2009. Magnetite magnetosome and fragmental chain formation of Magnetospirillum magneticum AMB-1: transmission electron microscopy and magnetic observations. Geophys J Int 177:33–42. https://doi.org/10.1111/j.1365-246X.2009.04043.x.

2. Cornell RM, Schwertmann U. 2003. The Iron Oxides: Structure, Properties, Reactions, Occurrences and Uses. John Wiley & Sons.

3. Jones SR, Wilson TD, Brown ME, Rahn-Lee L, Yu Y, Fredriksen LL, Ozyamak E, Komeili A, Chang MCY. 2015. Genetic and biochemical investigations of the role of MamP in redox control of iron biomineralization in Magnetospirillum magneticum. Proc Natl Acad Sci U S A 112:3904–3909. https://doi.org/10.1073/pnas.1417614112.

Supplementary figures

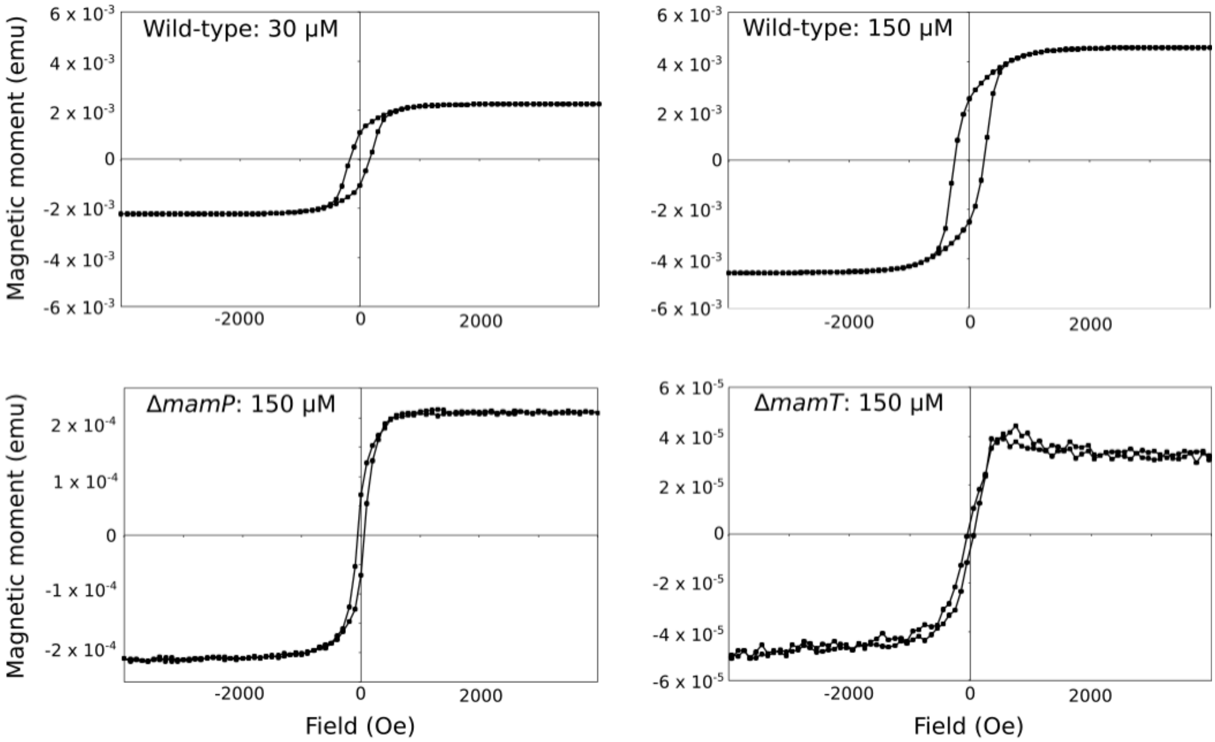

FIG S1 Hysteresis loops acquired from whole bacterial cultures, corresponding to wild-type AMB-1 cultivated under low- and high-iron conditions, and the Δ*mamP* and Δ*mamT* strains. Note the different y-axes.

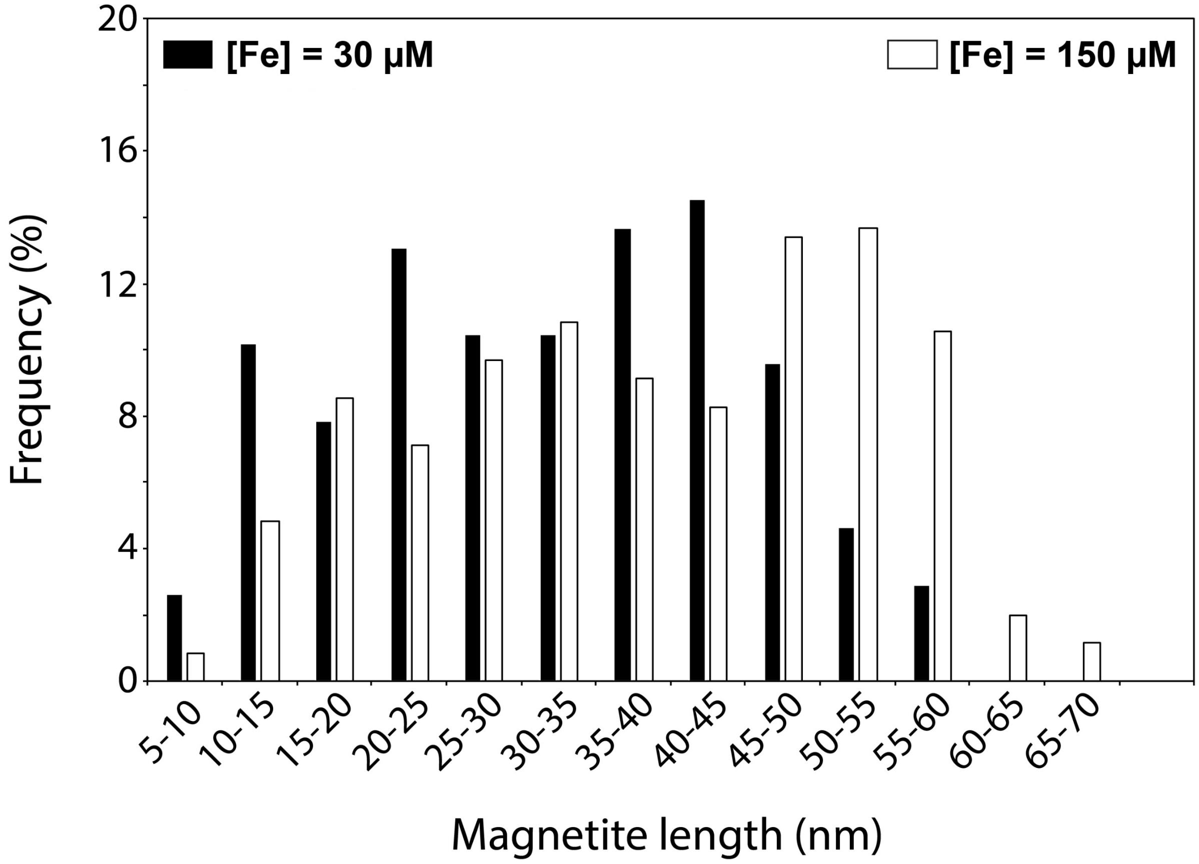

FIG S2 Distribution of magnetite length produced in wild-type AMB-1 cultivated for three days with iron at 30 (black bars) or 150 μM (white bars).

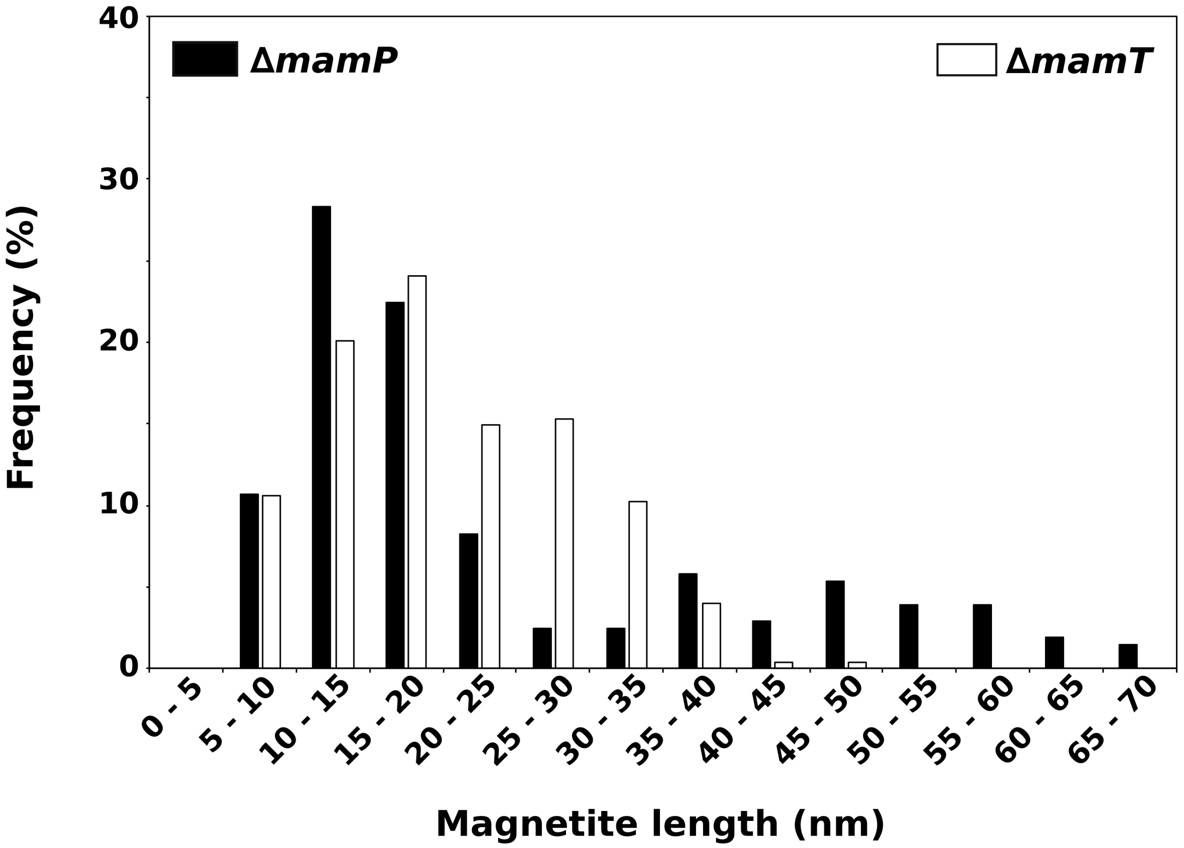

FIG S3 Distribution of magnetite length produced in Δ*mamP* (black bars) and 150 Δ*mamT* (white bars) AMB-1.

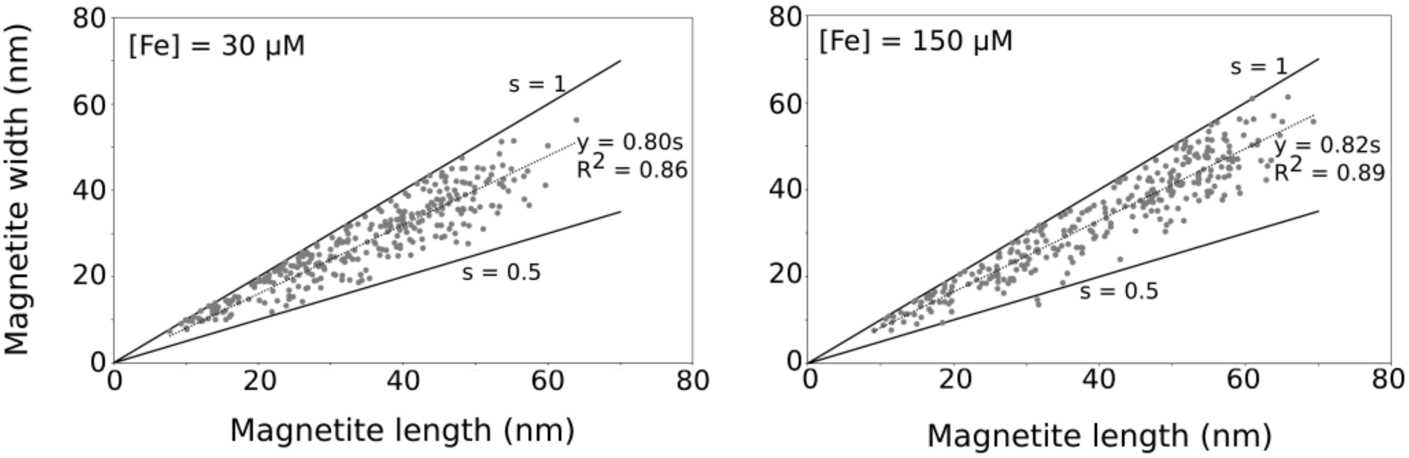

FIG S4 Magnetite width represented as a function of magnetite length in AMB-1 cultivated with iron at 30 (left) or 150 (right) µM. Assuming a constant shape factor (s) (Eq. S1), a linear relationship exists between width and length. Data are represented with the best linear fit, as well as two linear fits for a given shape factor of 1 and 0.5.

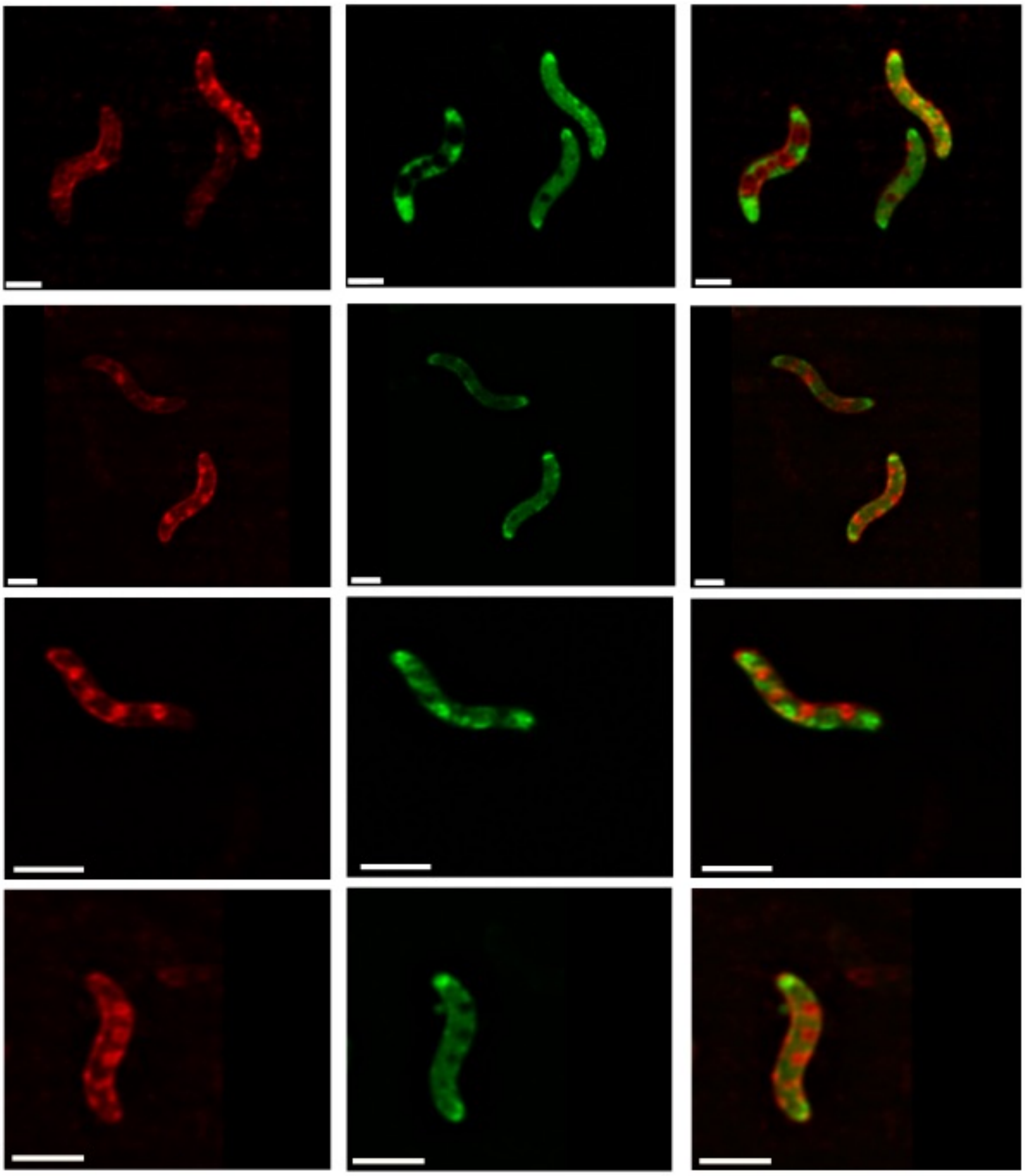

FIG S5 Red (left panels), green (center panels), and merged (right panels) fluorescence images of wild-type AMB-1 incubated with FIP-1 for 90 min. Scale bars = 1 μm.

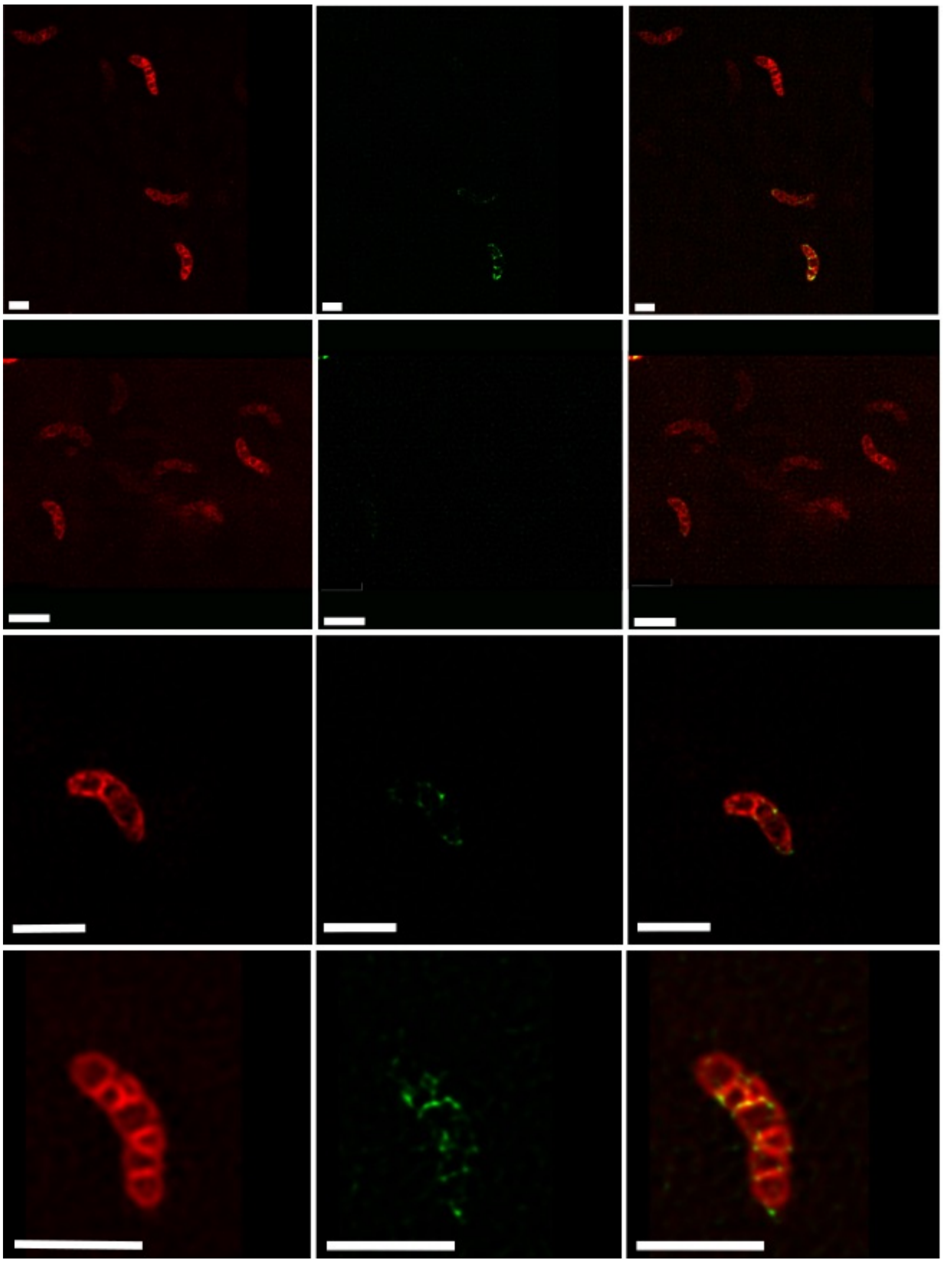

FIG S6 Red (left panels), green (center panels), and merged (right panels) fluorescence images of ΔMAI AMB-1 incubated with FIP-1 for 90 min. Scale bars = 1 μm.

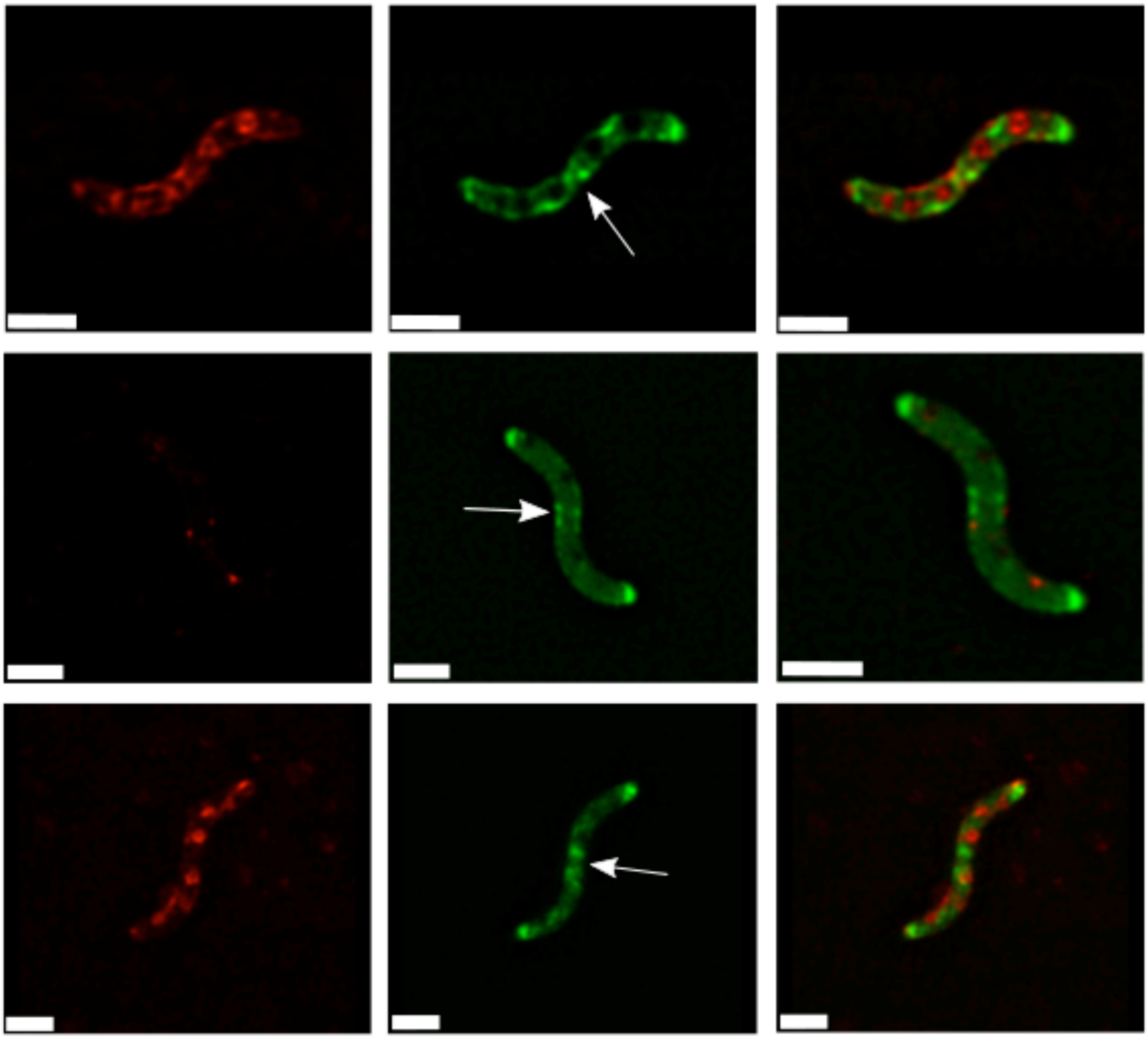

FIG S7 Red (left panels), green (center panels) and merged (right panels) fluorescence images of dividing wild-type cells incubated with FIP-1 for 90 min. Arrows point at higher green fluorescence signal in the cell that may be located at the septum location during cell division. Scale bars = 1 µm.

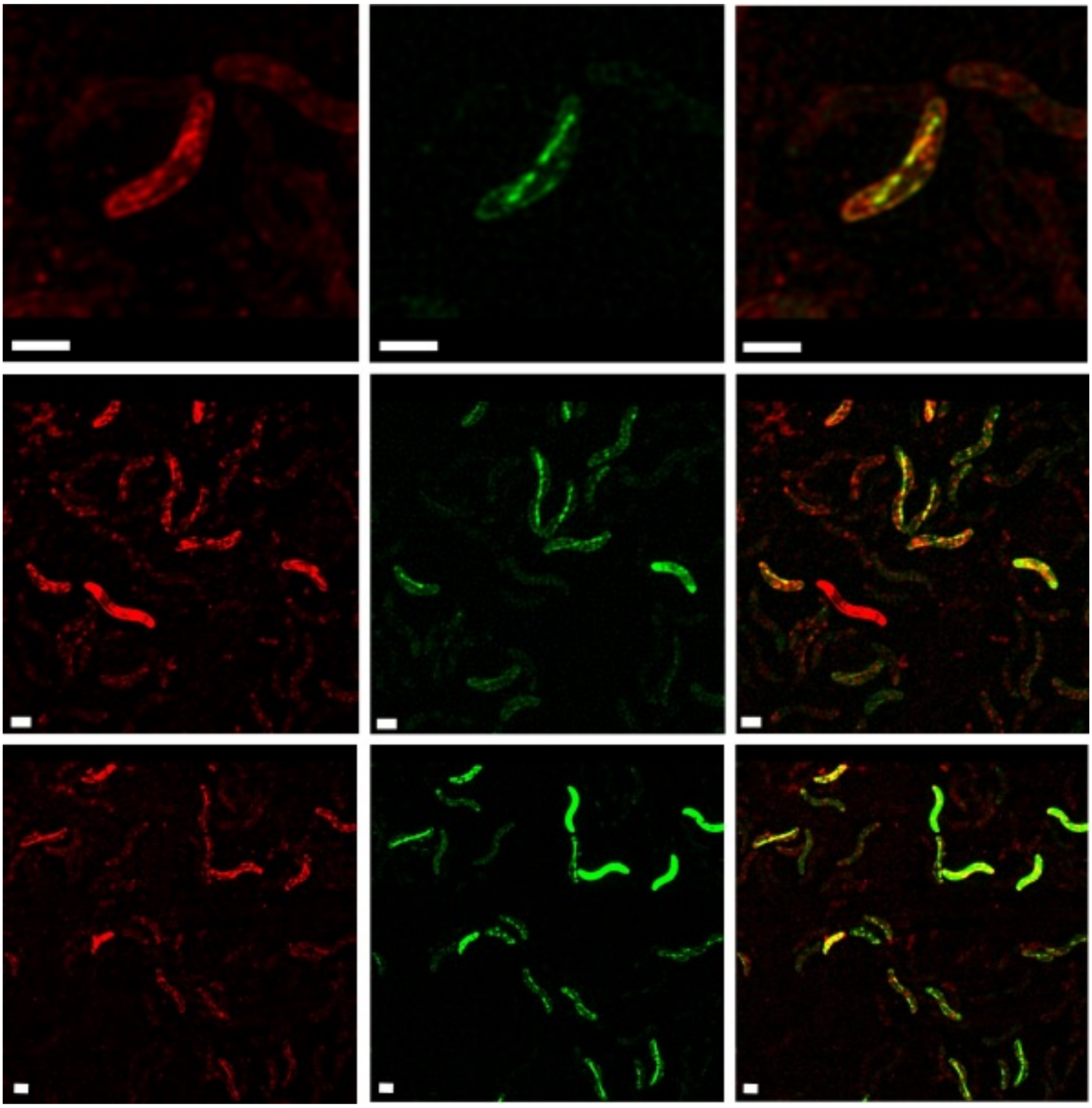

FIG S8 Red (left panels), green (center panels) and merged (right panels) fluorescence images of wild-type AMB-1 incubated with FIP-1 for 180 min. Scale bars = 1 μm.

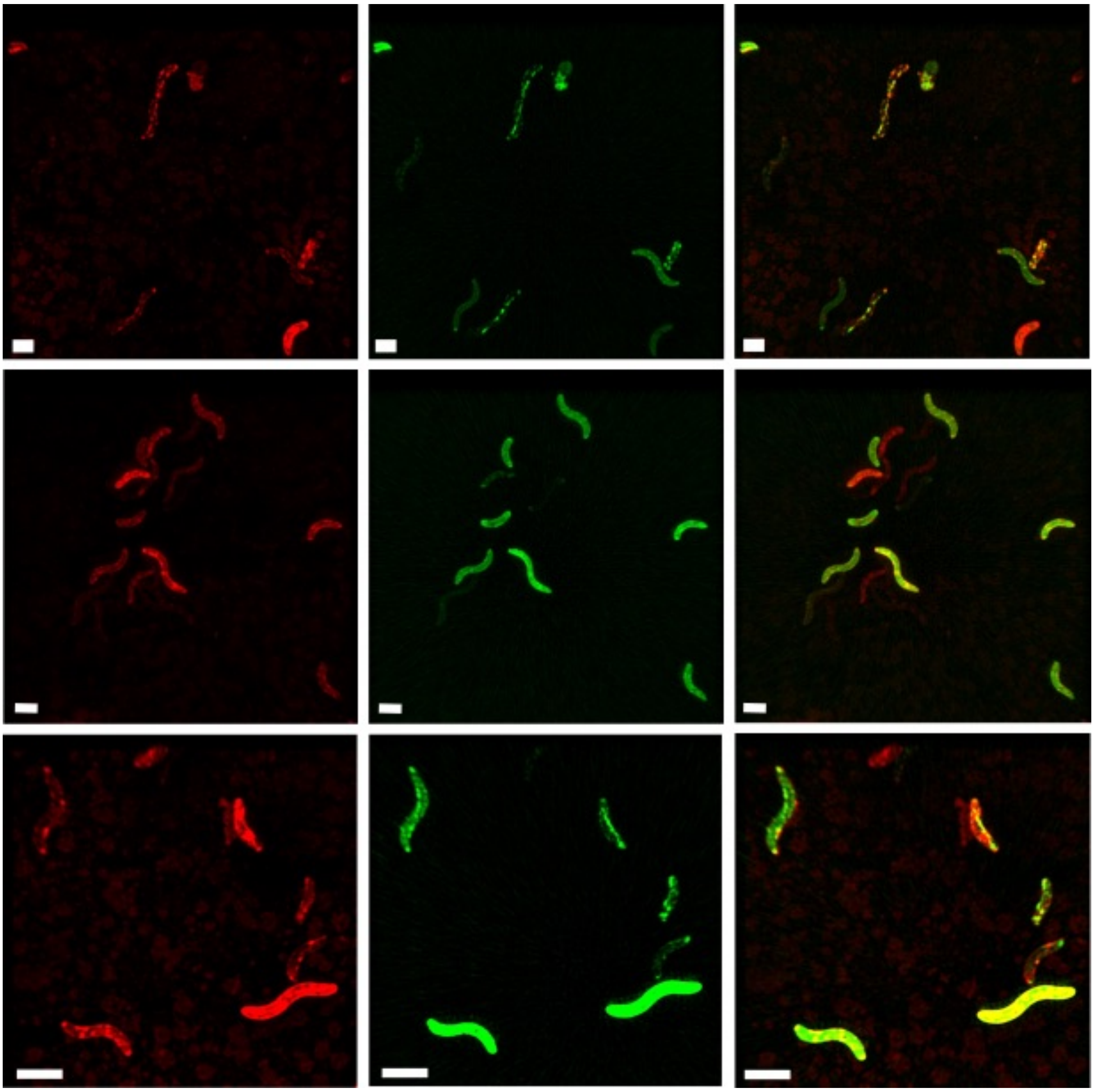

FIG S9 Red (left panels), green (center panels) and merged (right panels) fluorescence images of Δ*mamP* AMB-1 incubated with FIP-1 for 180 min. Scale bars = 2 μm.

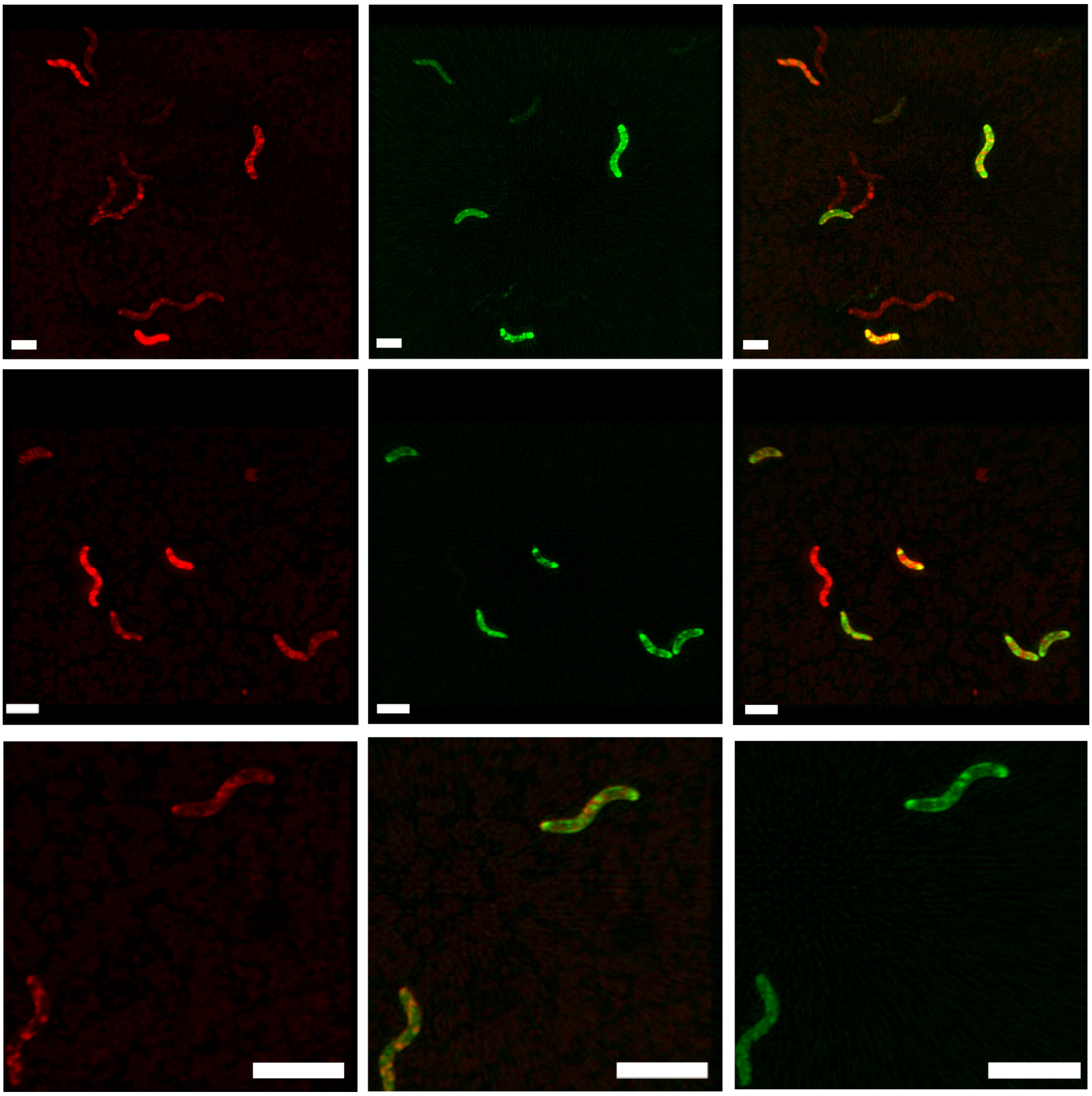

FIG S10 Red (left panels), green (center panels) and merged (right panels) fluorescence images of Δ*mamT* AMB-1 incubated with FIP-1 for 180 min. Scale bars = 2 μm.

Supplementary tables

Table S1 Iron mass balance in AMB-1 wild-type cultures.

Mass of iron in the initial medium (mg)	Mass of iron in the final medium (mg)	Mass of iron in bacteria (mg)	Mass of iron leaked outside of bacteria (mg)	Iron recovery (%)
2.31	2.20	0.06	$0.28\ 10^{-3}$	96
2.31	2.15	0.07	$0.17\ 10^{-3}$	96
2.37	2.31	0.05	$0.27\ 10^{-3}$	100
2.27	2.11	0.08	$0.23\ 10^{-3}$	97

Table S2 Magnetite crystal parameters measured from electron microscopy observations in wild-type (cultivated at either 30 or 150 µM of iron), Δ*mamP* and Δ*mamT* AMB-1 strains. Errors indicate 1 SD.

	**Wild-type (30 µM of iron)**	**Wild-type (150 µM of iron)**	**Δ*mamP***	**Δ*mamT***
Mean magnetite length (nm)	32.03	38.46	24.13	20.2
Width / length ratio	0.81	0.83	0.72	0.68
Number of crystals per cell	17.20 ± 4	20.75 ± 6	9.00 ± 3.57	21.00 ± 6.53
Number of crystals analyzed	344	351	205	274

References

Cornell RM, Schwertmann U. 2013. The iron oxides: structure, properties, reactions, occurrences and uses, second edition. Wiley-VCH Verlag GmbH & Co. KGaA.

Jones SR, Wilson TD, Brown ME, Rahn-Lee L, Yu Y, Fredriksen LL, Ozyamak E, Komeili A, Chang MCY. 2015. Genetic and biochemical investigations of the role of MamP in redox control of iron biomineralization in *Magnetospirillum magneticum*. Proc Natl Acad Sci USA 112: 3904-3909. https://doi.org/10.1073/pnas.1417614112.

Li J, Pan Y, Chen G, Liu Q, Tian L, Lin W. 2009. Magnetite magnetosome and fragmental chain formation of *Magnetospirillum magneticum* AMB-1: transmission electron microscopy and magnetic observations. Geophys J Int 177: 33-42. https://doi.org/10.1111/j.1365-246X.2009.04043.x.
